# Supplementary material for: A multiplex, prime editing framework for identifying drug resistance variants at scale
Source: Cell Genom. 2026 Feb 20;6(5):101167. doi: 10.1016/j.xgen.2026.101167 (PMC13174216; doi:10.1016/j.xgen.2026.101167)
Supplement: Document S1. Figures S1–S11 [file mmc1.pdf]

Cell Genomics, Volume *6*

Supplemental information

A multiplex, prime editing framework

for identifying drug resistance variants at scale

Florence M.C. Abadie, Chase C. Suiter, Nahum T. Smith, Riza M. Daza, Mary C. Rominger, Phoebe Parrish, Troy A. McDiarmid, Jean-Benoît Lalanne, Beth Martin, Diego Calderon, Amira Ellison, Alice H. Berger, Jay Shendure, and Lea M. Starita

Supplemental Information

A multiplex, prime editing framework for identifying drug resistance variants at scale

Florence M. C. Abadie[1,2,†,*], Chase C. Suiter[1,2,3,†], Nahum T. Smith[4], Riza M. Daza[1], Mary C. Rominger[5], Phoebe Parrish[1,5], Troy A. McDiarmid[1], Jean-Benoît Lalanne[1], Beth Martin[1], Diego Calderon[1,6], Amira Ellison[7], Alice H. Berger[1,5,*], Jay Shendure[1,2,4,8,9,*], Lea M. Starita[1,4,10*]

Affiliations:
1. Department of Genome Sciences, University of Washington, Seattle, WA, 98195, USA
2. Seattle Hub for Synthetic Biology, Seattle, WA, 98109, USA
3. Molecular and Cellular Biology Program, University of Washington, Seattle, WA, 98195, USA
4. Brotman Baty Institute for Precision Medicine, Seattle, WA, 98195, USA
5. Fred Hutchinson Cancer Research Center, Seattle, WA, 98109, USA
6. Bioengineering and Therapeutic Sciences, University of California San Francisco, CA, 94143, USA
7. Department of Molecular and Human Genetics, Baylor College of Medicine, Houston, TX, 77030, USA
8. Howard Hughes Medical Institute, Seattle, WA, 98195, USA
9. Allen Discovery Center for Cell Lineage Tracing, Seattle, WA, 98109, USA
10. Lead contact

† These authors contributed equally to this work

* Correspondence to F.M.C.A. (florence.chardon@alleninstitute.org), A.H.B. (ahberger@fredhutch.org), J.S. (shendure@uw.edu), or L.S. (lstarita@uw.edu)

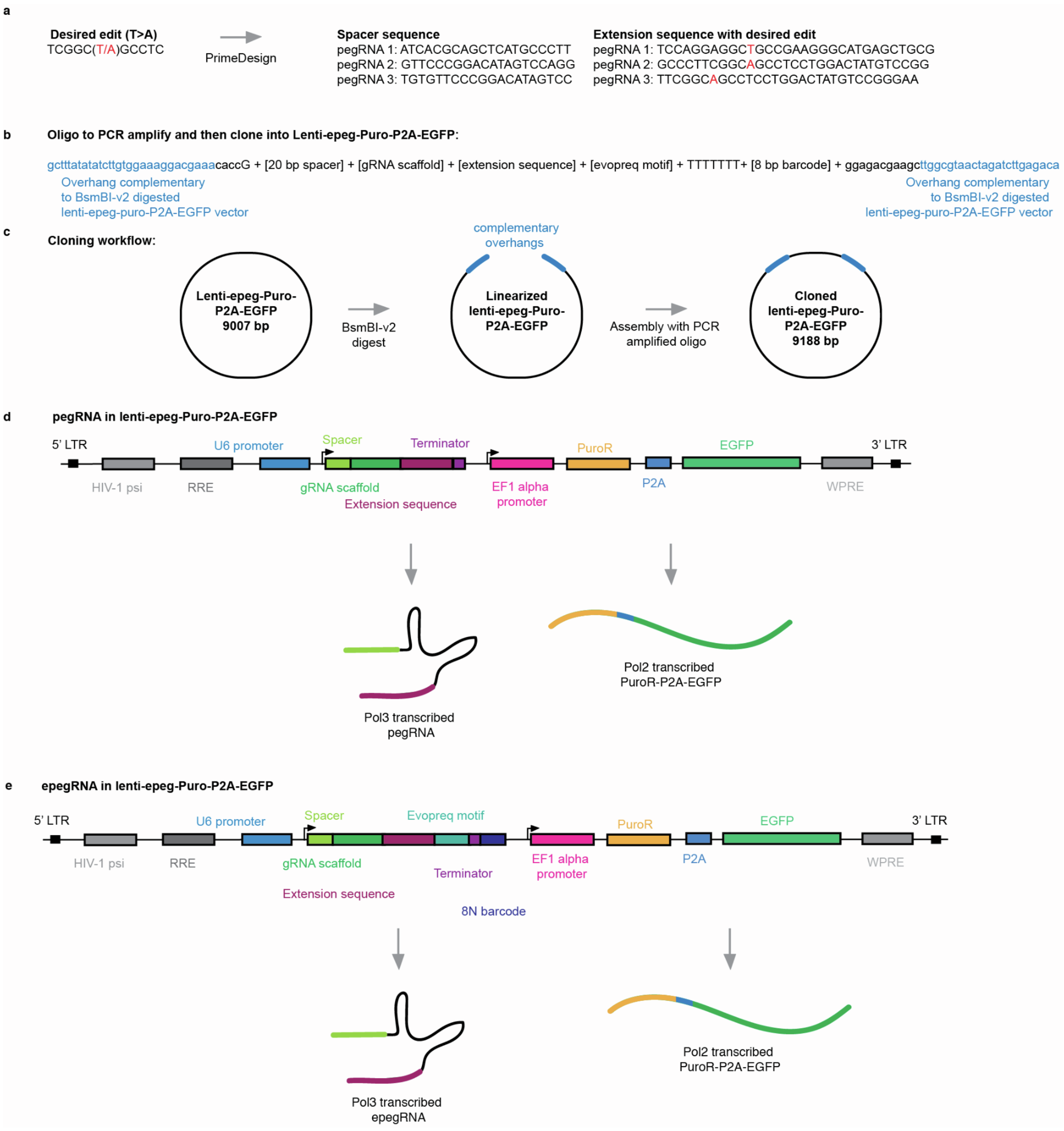

Figure S1 | pegRNA design, cloning, and expression vector components. Related to STAR methods.

a) Schematic of workflow to design pegRNAs with PrimeDesign[57]. Desired edit is in red. **b)** DNA sequence (oligo) to amplify and clone into lenti-epeg-Puro-P2A-EGFP. **c)** Schematic of cloning workflow. Lenti-epeg-Puro-P2A-EGFP is digested with BsmBI-v2, and the PCR amplified oligo from **b)** is assembled with the linearized vector via a Gibson assembly reaction. **d)** Schematic of the lenti-epeg-Puro-P2A-EGFP vector with a pegRNA cloned into it. **e)** Schematic of the lenti-epeg-Puro-P2A-EGFP vector with an epegRNA cloned into it (the epegRNA contains an evopreq RNA stabilizing motif and an 8N barcode sequence 3' of the terminator sequence).

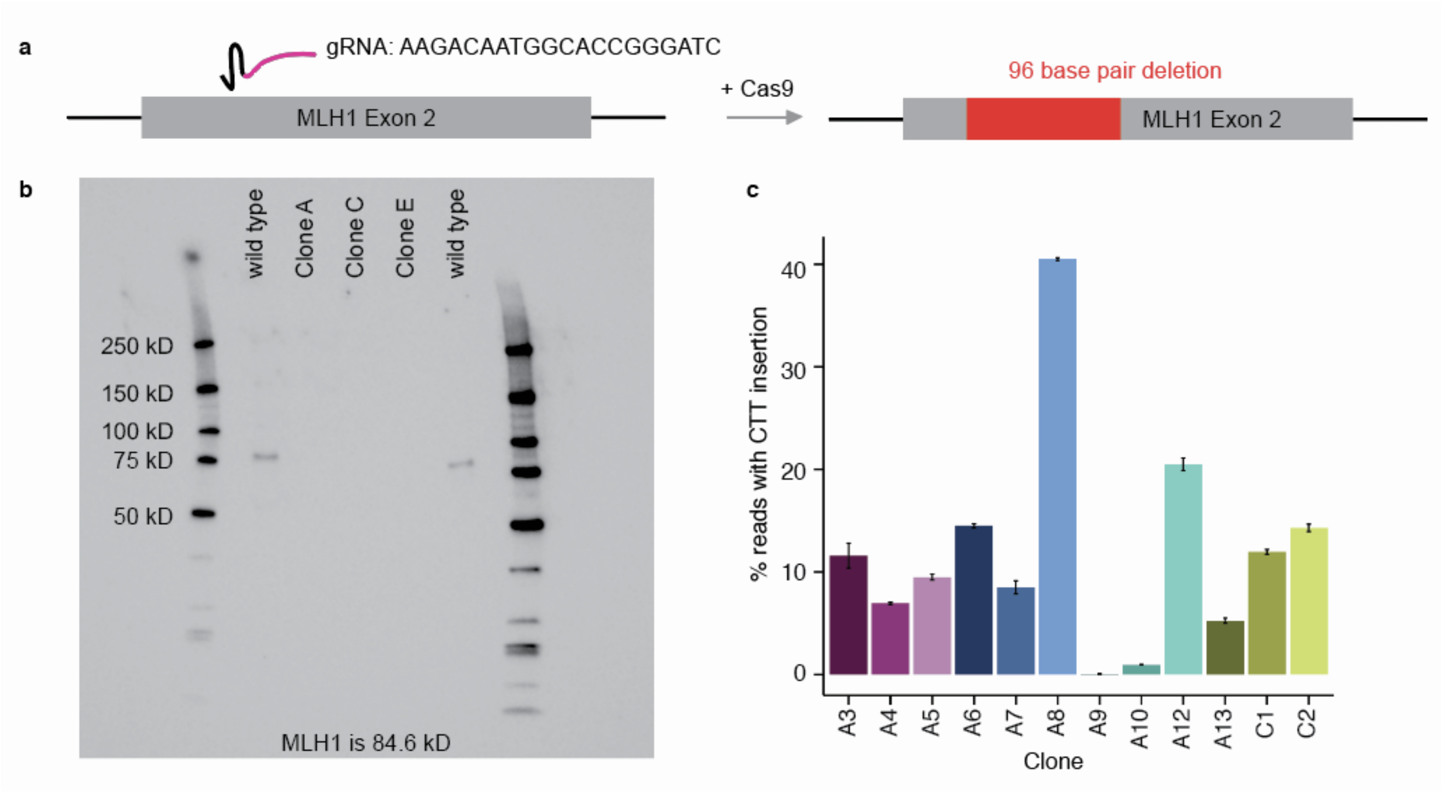

Figure S2 | Improvements to prime editing efficiency via an *MLH1* knockout. Related to STAR methods.

a) A single gRNA (in the pSpCas9(BB)-2A-Puro vector) targeting exon 2 in *MLH1* was transfected into PC-9. This knockout led to a 96 base pair deletion in both copies of *MLH1* in PC-9 cells. **b)** Western blot analysis of three PC-9 knockout clones. All three clones (clones A, C, and E) show complete loss of the MLH1 protein. Wild type cells were run in parallel (lanes 1 and 5) and show presence of the MLH1 protein. **c)** 12 monoclonal *MLH1*ko-PEmax-PC-9 cell lines were tested for insertion efficiency of a trinucleotide CTT insertion at the *HEK3* locus.

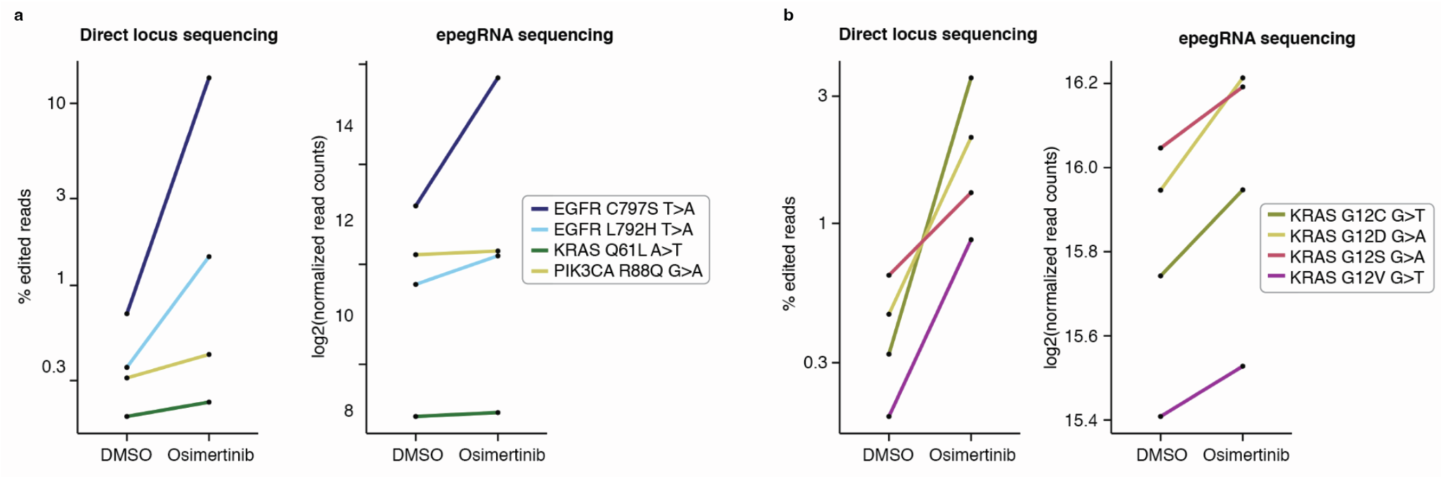

Figure S3 | Side-by-side sequencing of genomic loci and epegRNAs targeting those loci. Related to Figure 2.

a) Left: Percent edited reads by direct locus sequencing results in the no drug (DMSO) and osimertinib drug treatment arms. Right: log2 normalized read counts 339 of the epegRNA that programmed the specified edits. **b)** Same as a) but for a different set of targets.

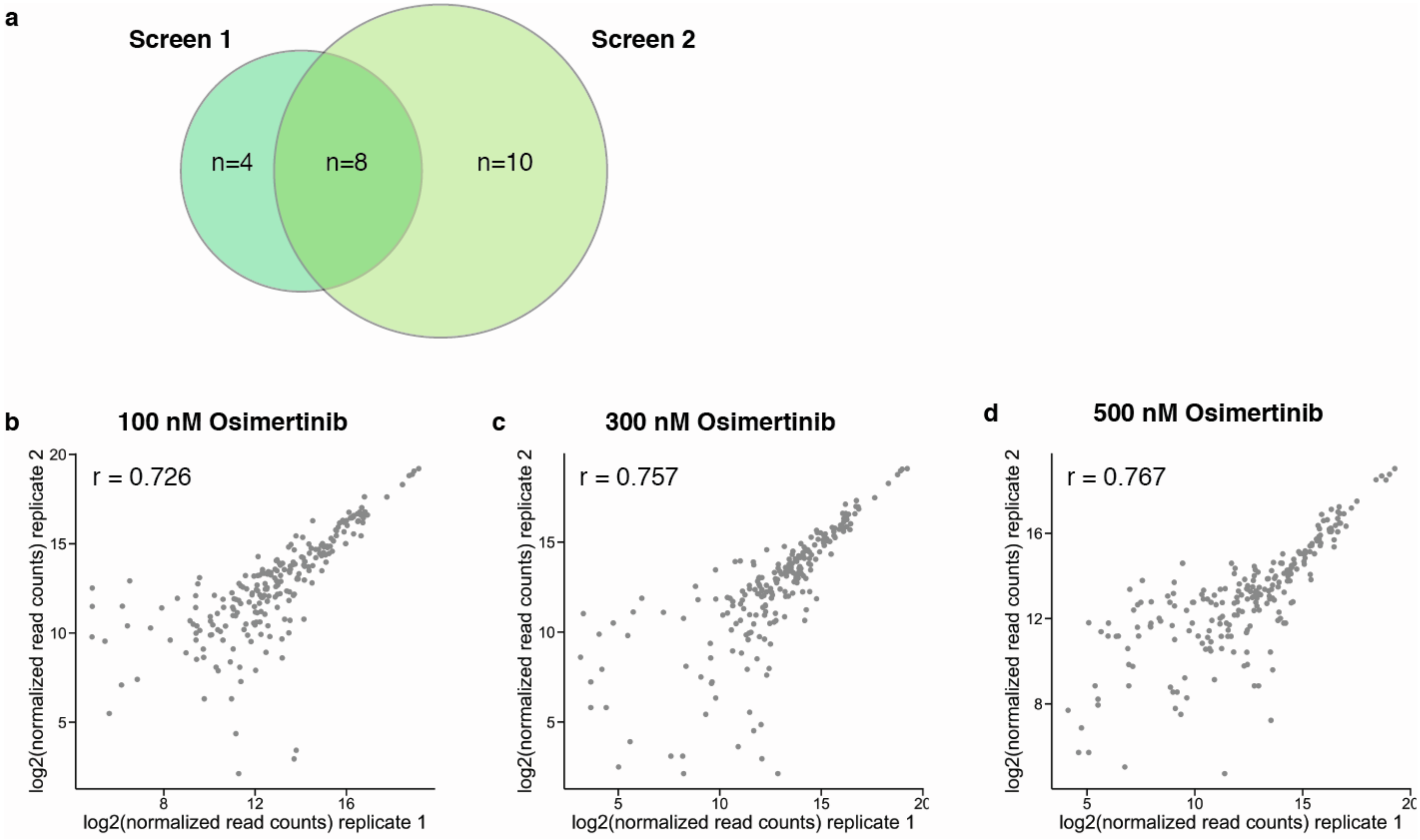

Figure S4 | Overlap of resistant variants between 121 epegRNA screens and replicate correlation of second 121 epegRNA screen. Related to Figure 2.

a) Overlap of hits between the first and second 121 epegRNA screens. **b)** Replicate correlation shown for 121 epegRNA screen at 100 nM osimertinib harvested at seven timepoints (days 3, 7, 10, 14, 17, 21, and 24). Each data point represents a single epegRNA at a single timepoint. **c)** Replicate correlation shown for 121 epegRNA screen at 300 nM osimertinib harvested at seven timepoints (days 3, 7, 10, 14, 17, 21, and 24). Each data point represents a single epegRNA at a single timepoint. **d)** Replicate correlation shown for 121 epegRNA screen at 500 nM osimertinib harvested at seven timepoints (days 3, 7, 10, 14, 17, 21, and 24). Each data point represents a single epegRNA at a single timepoint.

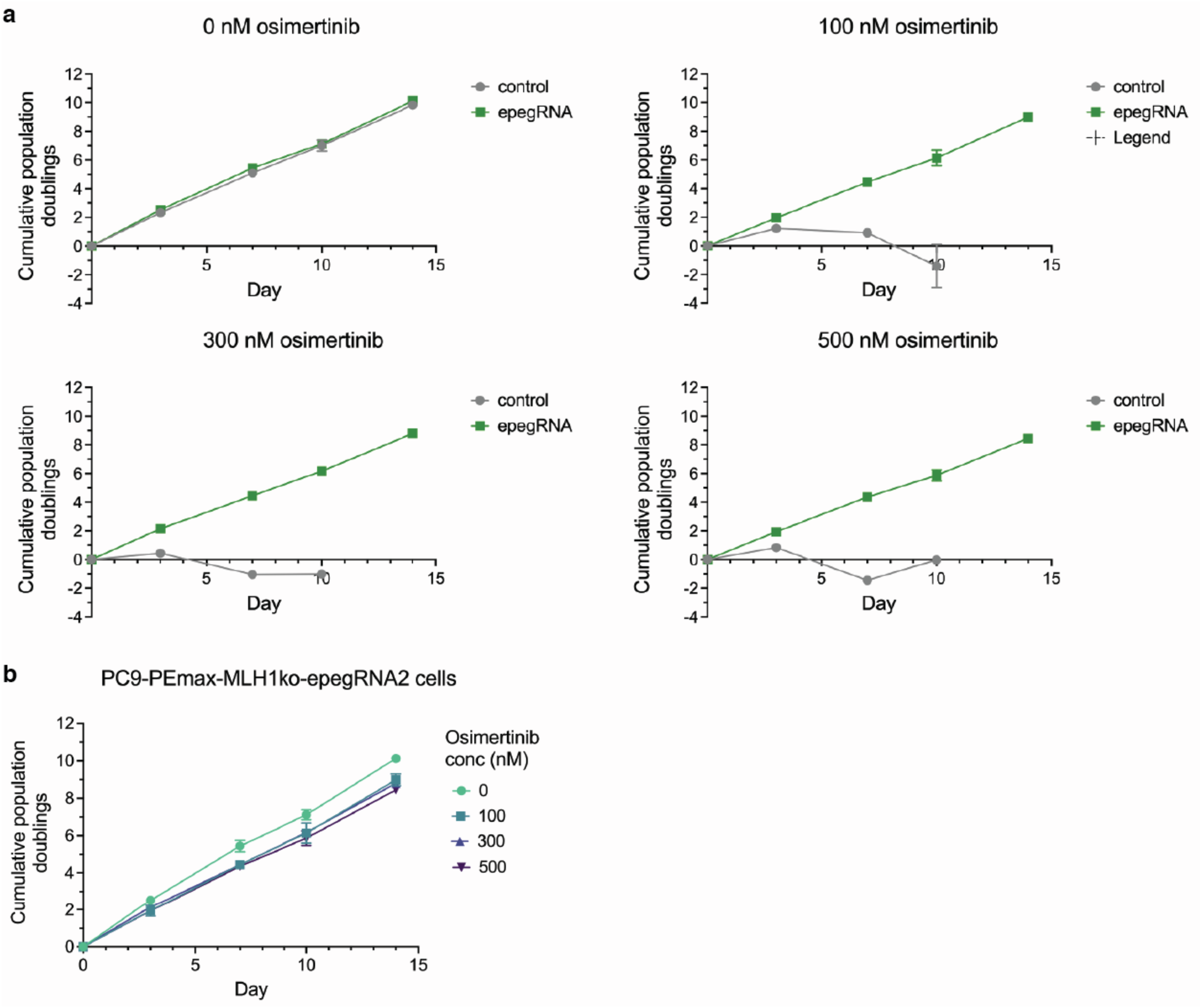

Figure S5 | PC-9 cell drug dosing experiments with varying concentrations of osimertinib. Related to STAR methods.

a) PC-9 cells with no integrated pegRNA (“control”) or a pegRNA programming the EGFR C797S T>A osimertinib resistance mutation (“epegRNA”). Cells were treated with 0, 100, 300, and 500 nM osimertinib and cell population doublings were tracked over a period of 14 days. Data shown are the mean ± standard deviation of two biological replicates. **b)** Same data as in **a)**. Cell population doublings of osimertinib resistant, EGFR C797S T>A harboring cells in 0, 100, 300, and 500 nM osimertinib.

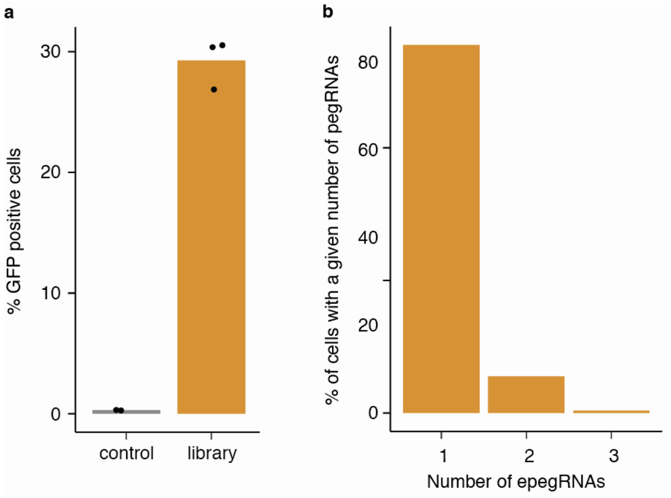

Figure S6 | Lentiviral transduction of 3,825 epegRNA library for scaled screen. Related to STAR methods.

a) *MLH1*ko-PEmax-PC-9 cells were analyzed by fluorescence activated cells sorting (FACS) to analyze the percentage of GFP+ cells following lentiviral transduction of the epegRNA library (GFP is expressed off the lentiviral epegRNA vector). Control cells were not transduced, and the library cells were transduced with the epegRNA library. The three data points represent three independent transduction replicates. **b)** Plot showing the percentage of cells harboring 1, 2, and 3 epegRNAs based off of the achieved MOI (~0.35) assuming a Poisson distribution of the number of integrations per cell.

a

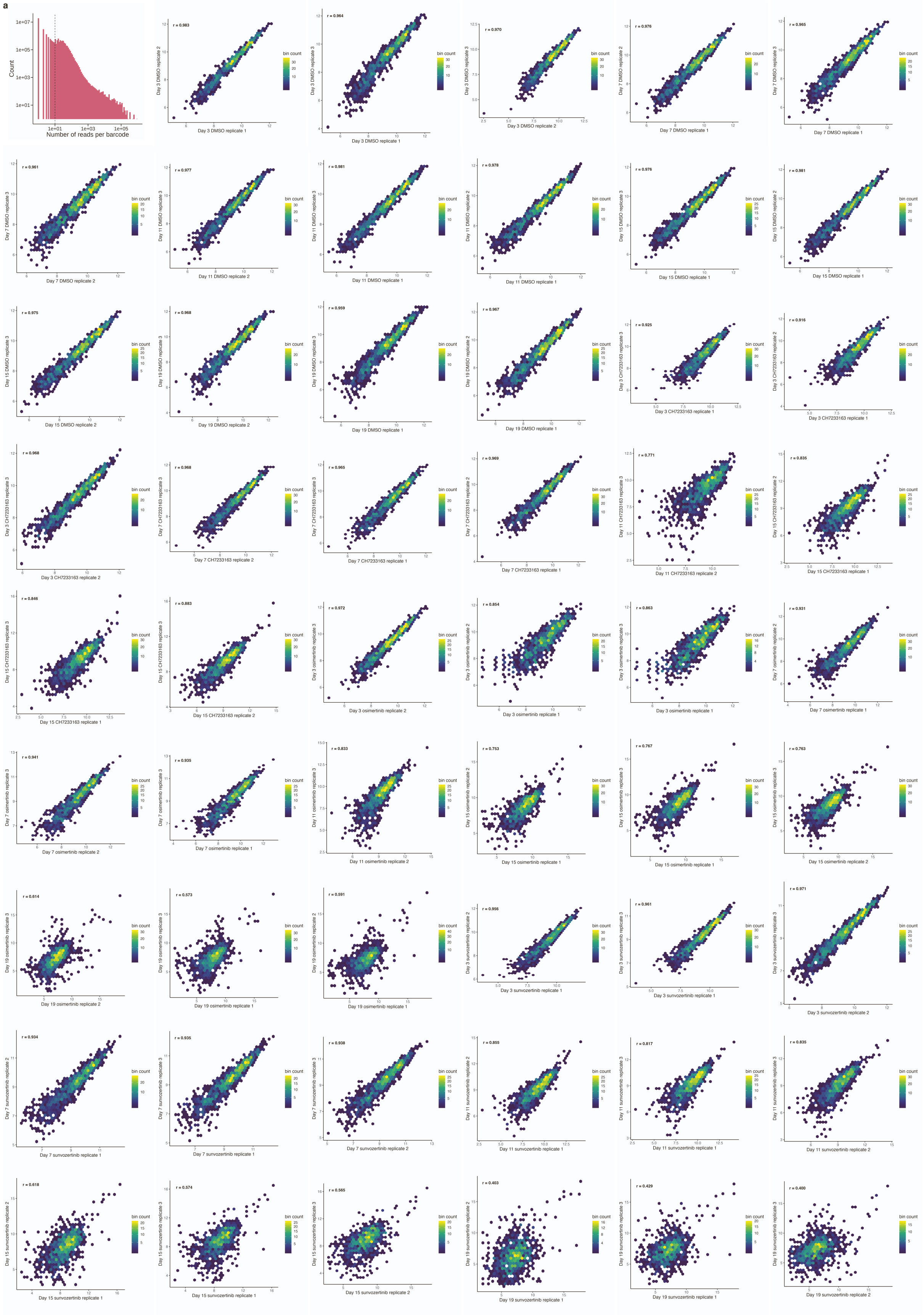

Figure S7 | Barcode read count cutoff and replicate correlations in scaled screen. Related to STAR methods.

a) Histogram showing the number of sequencing reads per barcode. A read cutoff of 10 reads per barcode was used for all analyses. **b)** Replicate correlation plots of log2(normalized read counts per edit ID) of three independent transduction replicates in the three drug screens.

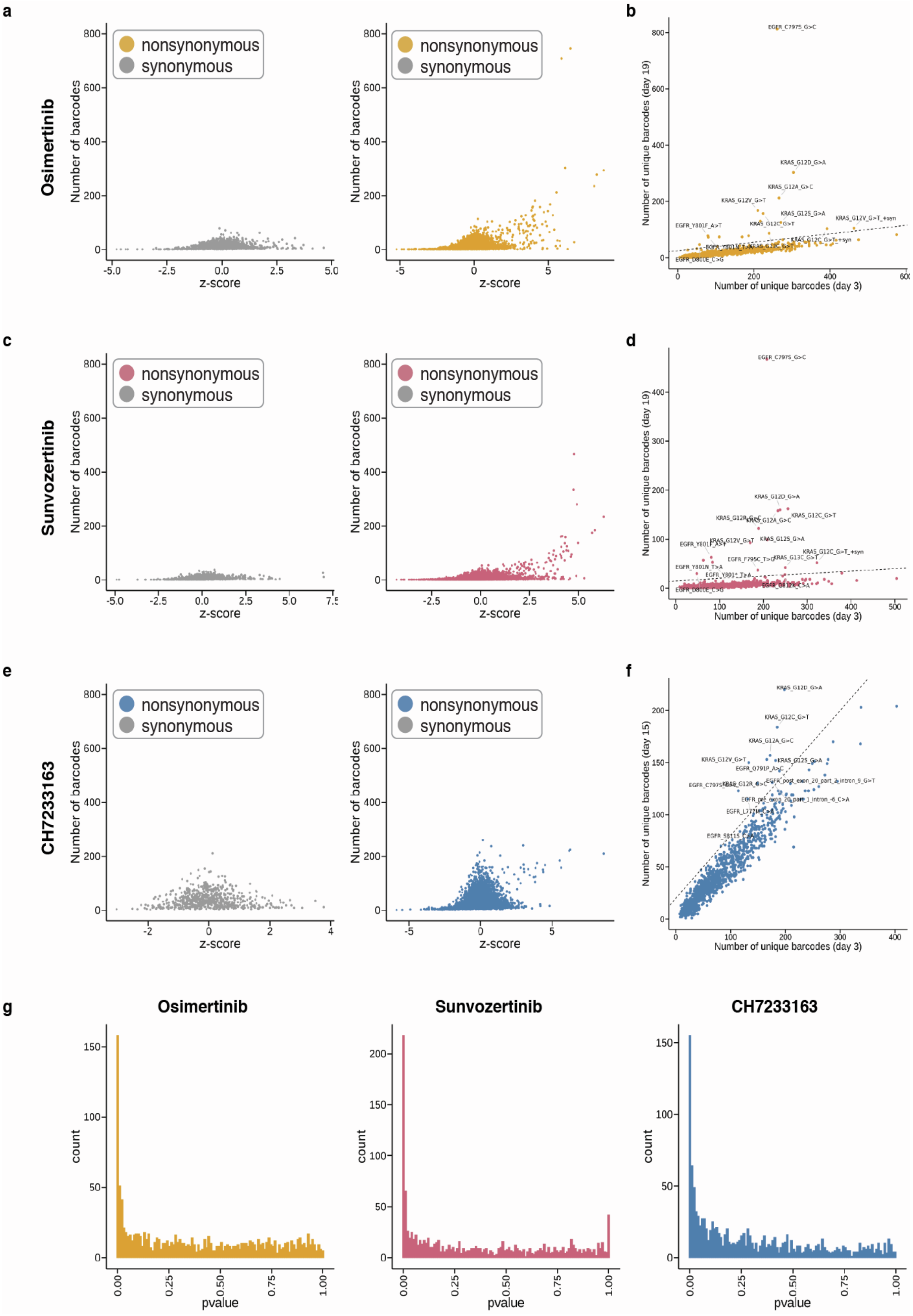
a
b
c
d
e
f
g
Osimertinib
Sunvozertinib
CH7233163
nonsynonymous
synonymous
Number of barcodes
z-score
Number of unique barcodes (day 19)
Number of unique barcodes (day 3)
Number of unique barcodes (day 15)
EGFR_C797S_G>C
KRAS_G12D_G>A
KRAS_G12A_G>C
KRAS_G12V_G>T
KRAS_G12S_G>A
KRAS_G12C_G>T
count
pvalue

Figure S8 | Z-score, barcode count, and p-value statistics from 3,825 epegRNA drug screens. Related to Figure 3.

a) Z-score and barcode counts plotted for day 15 and day 19 data (combined) for all three replicates for the osimertinib screen. Left: synonymous variants, right: nonsynonymous variants. **b)** Unique barcode count correlation plot between day 3 and day 19 of the osimertinib screen. Variants that fall above the diagonal (y = 0.15x + 25) are labeled. **c)** Z-score and barcode counts plotted for day 15 and day 19 data (combined) for all three replicates for the sunvozertinib screen. Left: synonymous variants, right: nonsynonymous variants. **d)** Unique barcode count correlation plot between day 3 and day 19 of the sunvozertinib screen. Variants that fall above the diagonal (y = 0.05x + 15) are labeled. **e)** Z-score and barcode counts plotted for day 15 and day 19 data (combined) for all three replicates for the CH7233163 screen. Left: synonymous variants, right: nonsynonymous variants. **f)** Unique barcode count correlation plot between day 3 and day 15 of the CH7233163 screen. Variants that fall above the diagonal (y = 0.6x + 20) are labeled. **g)** p-value distributions from DESeq2 differential pegRNA abundance testing for the three drug screens.

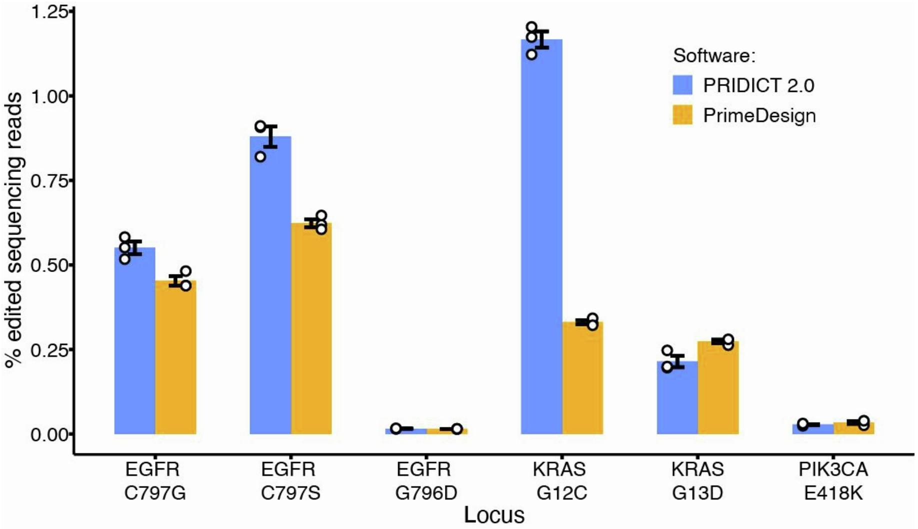

Figure S9 | Comparison of pegRNAs designed by PrimeDesign and PRIDICT 2.0. Related to STAR methods. Edit efficiencies of six different variants using pegRNAs designed with both PRIDICT 2.0 (blue) or PrimeDesign (yellow). Each bar represents the average of three replicates (denoted by points). Error bars represent ± one standard error of the mean.

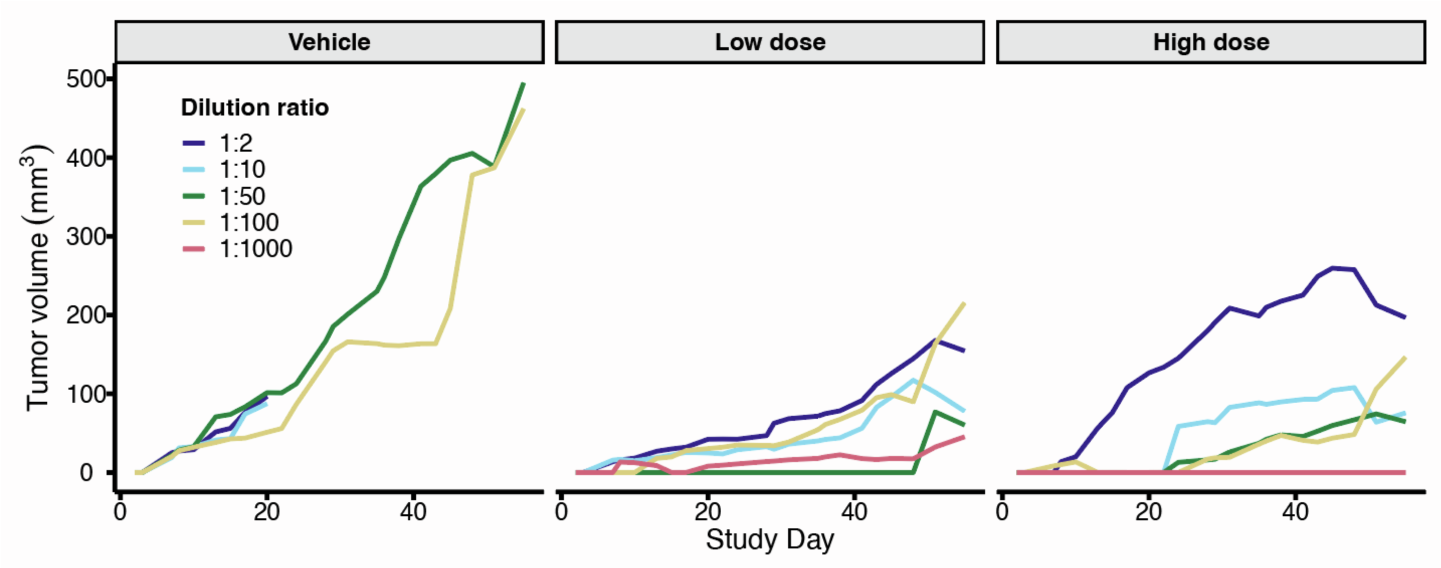

Figure S10 | Tumor volume over time in mice injected with mixtures of wild-type and EGFR C797S mutant PC-9 cells. Related to Figure 4.

Visualization of tumor volume over time in mice injected with mixtures of wild-type and EGFR C797S mutant PC-9 cells mixed at the indicated ratios. Plots are faceted by treatment group, including vehicle (DMSO control), low dose osimertinib (5 mg/kg) and high dose osimertinib (20 mg/kg).

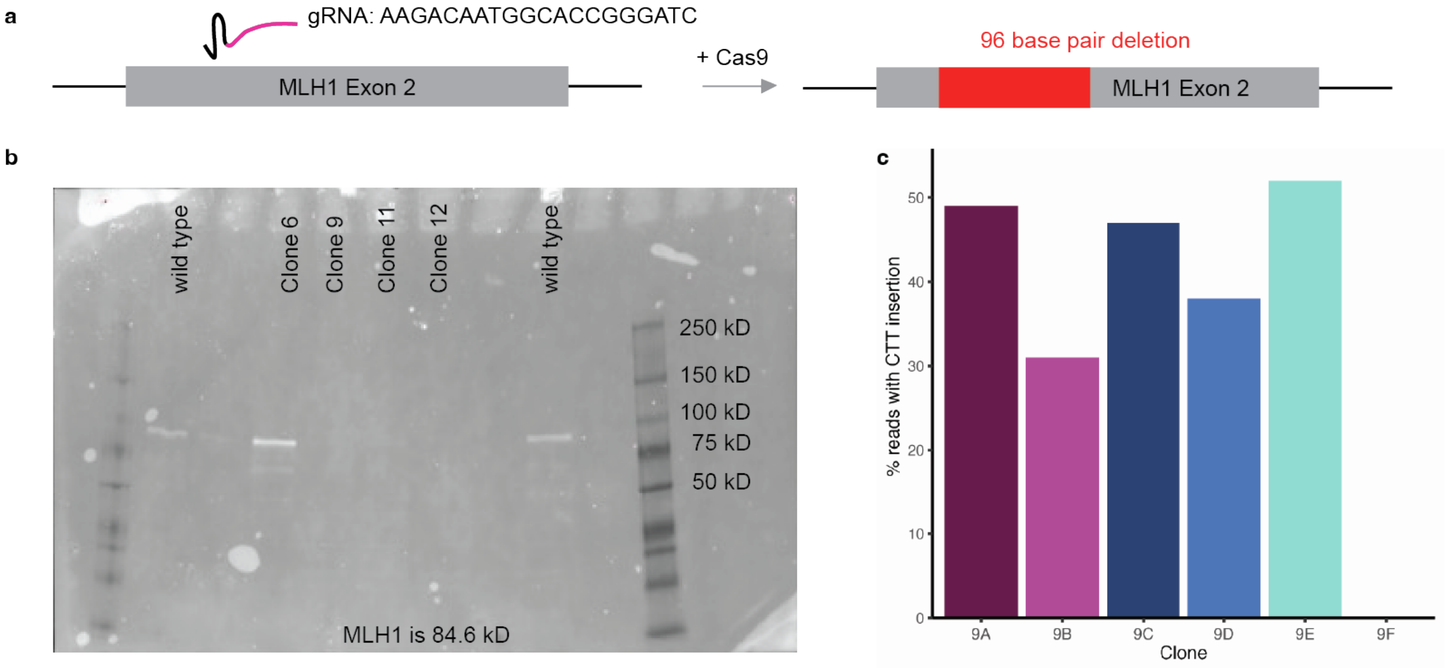

Figure S11 | Generation of an *MLH1* knockout A-375 cell line. Related to STAR methods.

a) A single gRNA (in the pSpCas9(BB)-2A-Puro vector) targeting exon 2 in *MLH1* was transfected into A-375 cells. This knockout leads to a 96 base pair deletion in both copies of *MLH1* in A-375 cells when successful. **b)** Western blot analysis of four A-375 knockout clones. Three of four clones (clones 9, 11, and 12) show complete loss of the MLH1 protein. Wild type cells were run in parallel (lanes 1 and 6) and show presence of the MLH1 protein. **c)** 6 monoclonal subclones of Clone 9 were tested for insertion efficiency of a trinucleotide CTT insertion at the *HEK3* locus. Clone 9E was used for all experiments.
