# Supplementary material for: A multiplex, prime editing framework for identifying drug resistance variants at scale
Source: Cell Genom. 2026 Feb 20;6(5):101167. doi: 10.1016/j.xgen.2026.101167 (PMC13174216; doi:10.1016/j.xgen.2026.101167)
Supplement: Document S2. Article plus supplemental information [file mmc22.pdf]

Cell Genomics

Article

A multiplex, prime editing framework for identifying drug resistance variants at scale

Graphical abstract

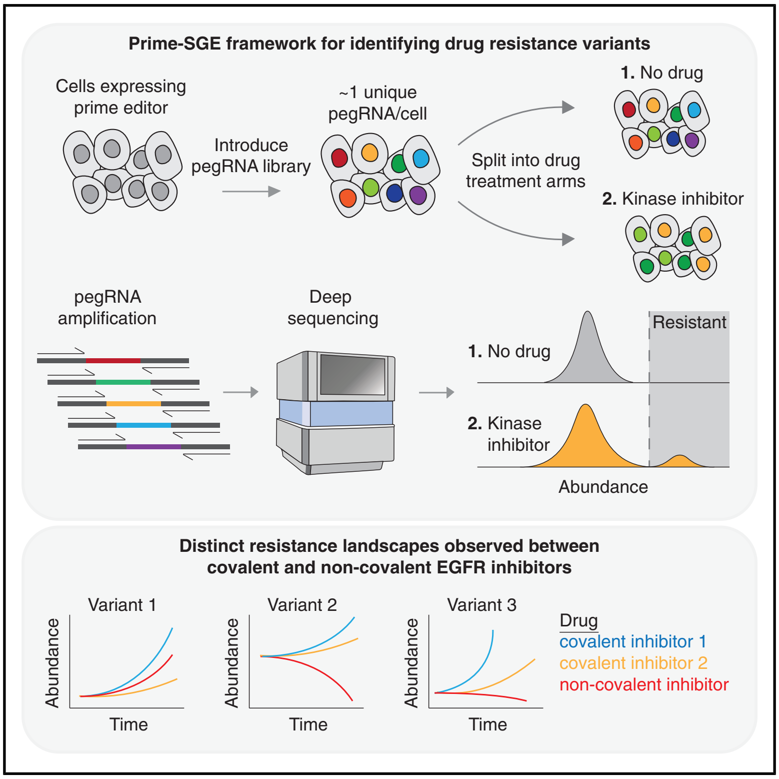

Highlights

- Prime-SGE enables scalable resistance screening in cancer cell lines
- A positive selection framework yields high-confidence inhibitor resistance hits
- Prime-SGE identifies both established and less-characterized resistance mutations
- Covalent and non-covalent EGFR inhibitors show distinct resistance landscapes

Authors

Florence M.C. Abadie, Chase C. Suiter, Nahum T. Smith, ..., Alice H. Berger, Jay Shendure, Lea M. Starita

Correspondence

florence.abadie@alleninstitute.org (F.M.C.A.),
ahberger@fredhutch.org (A.H.B.),
shendure@uw.edu (J.S.),
lstarita@uw.edu (L.M.S.)

In brief

Abadie and Suiter et al. introduce prime-SGE, a prime editing framework enabling multiplexed functional testing of thousands of single-nucleotide variants. Applying prime-SGE, they profile mutations across eight oncogenes for resistance to four EGFR tyrosine kinase inhibitors, demonstrating a scalable approach to identify and compare resistance variants across multiple inhibitor contexts.

Abadie et al., 2026, Cell Genomics *6*, 101167
May 13, 2026 © 2026 The Author(s). Published by Elsevier Inc.
https://doi.org/10.1016/j.xgen.2026.101167

CellPress

Cell Genomics

CellPress
OPEN ACCESS

Article

A multiplex, prime editing framework for identifying drug resistance variants at scale

Florence M.C. Abadie,[1,2,10,*] Chase C. Suiter,[1,2,3,10] Nahum T. Smith,[4] Riza M. Daza,[1] Mary C. Rominger,[5] Phoebe Parrish,[1,5] Troy A. McDiarmid,[1] Jean-Benoît Lalanne,[1] Beth Martin,[1] Diego Calderon,[1,6] Amira Ellison,[7] Alice H. Berger,[1,5,*] Jay Shendure,[1,2,4,8,9,*] and Lea M. Starita[1,4,11,*]

[1]Department of Genome Sciences, University of Washington, Seattle, WA 98195, USA
[2]Seattle Hub for Synthetic Biology, Seattle, WA 98109, USA
[3]Molecular and Cellular Biology Program, University of Washington, Seattle, WA 98195, USA
[4]Brotman Baty Institute for Precision Medicine, Seattle, WA 98195, USA
[5]Fred Hutchinson Cancer Center, Seattle, WA 98109, USA
[6]Bioengineering and Therapeutic Sciences, University of California, San Francisco, San Francisco, CA 94143, USA
[7]Department of Molecular and Human Genetics, Baylor College of Medicine, Houston, TX 77030, USA
[8]Howard Hughes Medical Institute, Seattle, WA 98195, USA
[9]Allen Discovery Center for Cell Lineage Tracing, Seattle, WA 98109, USA
[10]These authors contributed equally
[11]Lead contact
*Correspondence: florence.abadie@alleninstitute.org (F.M.C.A.), ahberger@fredhutch.org (A.H.B.), shendure@uw.edu (J.S.), lstarita@uw.edu (L.M.S.)
https://doi.org/10.1016/j.xgen.2026.101167

SUMMARY

CRISPR-based genome editing has revolutionized functional genomics, enabling thousands of perturbations to be concurrently assayed in single experiments. However, for methods such as saturation genome editing (SGE), which aims to generate and assay libraries of point mutations, a challenge is that only one region (e.g., one exon) is studied per experiment. Here, we describe prime-SGE, a prime editing-based framework in which libraries of specific point mutations are installed into genes throughout the genome and then functionally assessed by sequencing of prime editing guide RNAs (pegRNAs) rather than the mutations themselves. We apply prime-SGE in two cell lines to assay thousands of point mutations in eight oncogenes for their ability to confer drug resistance to four tyrosine kinase inhibitors. Our prime-SGE strategy, combined with ongoing improvements in prime editing efficiency, opens the door to efficient positive selection screens of large numbers of point mutations at locations throughout the genome.

INTRODUCTION

Resistance to targeted therapies is a major barrier to the successful treatment of many cancers, including non-small cell lung cancer.[1–3] Both *de novo* and acquired resistance to therapeutic small molecules can be caused by mutations in target genes that inhibit drug binding, such as with *EGFR* resistance mutations to covalent EGFR inhibitors. Alternatively, resistance can develop by activating bypass signaling through mutation or amplification of other oncogenes.[4] Pinpointing resistance mutations and developing novel drugs that circumvent them is crucial for continued progress in the treatment of cancer.[1] Mutations associated with resistance can be identified retrospectively in clinical specimens from repeat biopsies or circulating tumor DNA.[5–8] However, obtaining these specimens can be challenging,[9] and causation of resistance must still be verified with functional experiments.[10]

Ideally, resistance mutations would be identified prospectively as part of the clinical development of each drug.[1] At least three complementary approaches have been taken to model drug resistance *in vitro*. First, cells can be cultured in the presence of a drug to allow resistant mutations to arise spontaneously, outcompete other cells until they rise to an appreciable frequency, and be identified by whole-genome or targeted sequencing.[11] Second, wild-type and mutant versions of oncogenes or tumor suppressors can be overexpressed to measure their capacity to provide resistance.[12] Finally, deep mutational scans of open reading frames can identify all potential resistance mutations in candidate genes.[10,13,14] However, these approaches are limited in important ways. For example, evolutionary selections are subject to chance and will likely only identify a subset of potential resistance mutations, while the latter approaches query transgenes rather than mutations in the endogenous genome. An ideal approach would concurrently introduce and characterize vast numbers of potential resistance mutations in genes of interest in their endogenous genomic context. This would allow for upfront identification of resistance mutations to drugs during early-stage development and possible repurposing of agents that can overcome drug resistant variants.

To functionally assess single nucleotide variants or specific small insertions or deletions at scale via CRISPR, a powerful

© 2026 The Author(s). Published by Elsevier Inc.
This is an open access article under the CC BY license (http://creativecommons.org/licenses/by/4.0/).

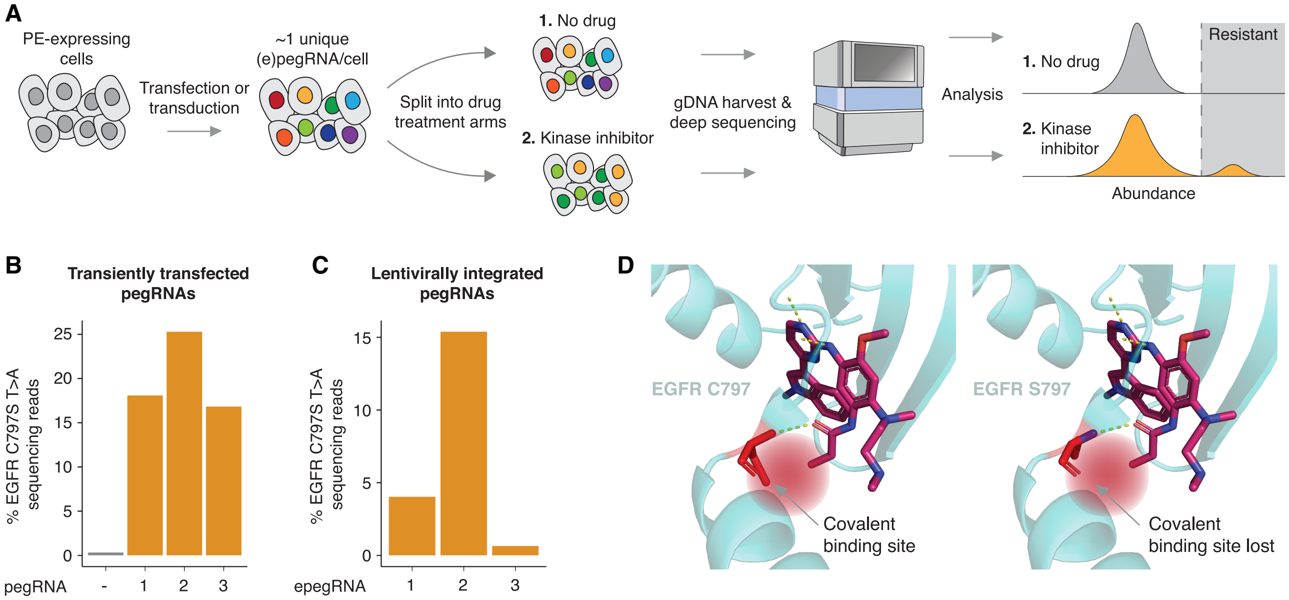

Figure 1. Prime editing of EGFR C797S confers resistance to osimertinib

(A) In prime-SGE, a library of prime editing gRNAs (pegRNAs) are lentivirally transduced into a pool of prime editor-expressing cells at a low multiplicity of infection (MOI) such that the majority of cells harbor a single, unique pegRNA. This pool of cells is then subjected to treatment with DMSO (no drug) or one of four kinase inhibitors and cultured for a period of ∼20 days (∼10 cell doublings). Integrated pegRNAs are amplified from genomic DNA and sequenced. Read counts are analyzed to determine the distribution of variant abundances across different treatment conditions.

(B and C) Wild-type PC-9 cells were transiently transfected or lentivirally transduced with a pegRNA or epegRNA programming the EGFR C797S T>A mutation, along with the prime editor enzyme. Osimertinib was added to the cells for 24 days, genomic DNA was harvested, and the EGFR locus was amplified and sequenced. Percentage of reads with the EGFR C797S T>A mutation after (B) transient transfection with three pegRNAs or (C) lentiviral transduction of three epegRNAs into PC-9 cells.

(D) Left: crystal structure of EGFR covalently bound with osimertinib when residue 797 is a cysteine (wild type; PDB: 4ZAU).[30] Right: crystal structure of EGFR no longer covalently binding osimertinib when residue 797 is mutated to a serine (C797S mutation).

approach is to leverage libraries of homology-directed repair (HDR) templates to install mutations of interest to a short region of interest (e.g., an exon). However, the major limitation of this strategy (also known as saturation genome editing [SGE][15–19]) is that only one region can be studied per experiment, both because the HDR template library relies on a locus-directing guide RNA (gRNA) and because deciphering which mutation was installed in which cell requires sequencing of the edited locus itself. As such, evaluating candidate resistance mutations in many exons of many genes in response to a panel of compounds via HDR-based SGE would be highly labor intensive. Saturating scans of multiple exons or genes are possible with base editing, but these lack precision (and range) with respect to which mutations are (or can be) introduced.[20,21]

Prime editing is a genome editing method that facilitates the precise installation of genetic variants by using a nicking Cas9 fused to a reverse transcriptase (prime editor) and a single prime editing gRNA (pegRNA).[22] Applying prime editing to assay the effects of single-nucleotide variants has two potentially major advantages over other CRISPR-based genome editing methods. First, it would facilitate multiplex experiments in which many mutations at many loci could be programmed within the context of a single experiment.[23] This is due to the unique design of the pegRNA, which specifies both the target site and programmed edit in a single molecule.[22] Second, it is expected to be more precise, as PE, which does not rely on disruptive DNA double-strand breaks, exhibits much lower rates of both target-proximal and off-target errors than CRISPR-Cas9-mediated HDR or base editing.[20,22]

Here, we present prime-SGE, a scalable, multiplex prime editing framework in which we introduce and assess thousands of precise edits simultaneously in oncogene-addicted cancer cell lines. We devised a positive selection strategy to overcome the low editing rate of prime editing and select for edits that provide resistance to various tyrosine kinase inhibitors (TKIs). In this framework, each cell is engineered to harbor, on average, a single pegRNA encoding a precise edit. This pool of cells is then subjected to TKI treatment or a vehicle control. Cells are harvested over a time course of 2–3 weeks.

In this proof of concept, we found that deep sequencing of the integrated pegRNAs allows for the identification of programmed mutations that give rise to drug resistant cells (Figure 1A). Specifically, prime-SGE was able to resolve well-characterized resistance mutations, such as EGFR C797S, KRAS G12C,[4] and BRAF V600E, and also identified potentially novel, previously uncharacterized resistance mutations. Our results, together with other recently reported methods that are complementary to prime-SGE,[24–29] suggest a promising future for multiplex prime editing for the functional analysis of genetic variants.

RESULTS

Prime editing of the osimertinib resistance mutation C797S in EGFR

As a first experiment, we sought to introduce a single, well characterized mutation to the *EGFR* gene that confers resistance to the small molecule TKI osimertinib[31] via prime editing and then ask whether we could detect its selection during an *in vitro* resistance screen. This mutation, a T to A base change at the first position of amino acid residue 797 of the EGFR open reading frame, changes the wild-type cysteine residue to a mutant serine residue. Osimertinib is a third-generation TKI that targets the ATP-binding pocket of EGFR by covalently binding the C797 residue[32] and is the current standard-of-care therapy for advanced-stage EGFR-mutant lung cancer.[33] The cysteine-to-serine change at position 797 blocks the binding of osimertinib and leads to drug resistance and poor survival outcomes.[31] We performed experiments in PC-9 cells, which are both addicted to EGFR signaling[34] and sensitive to the TKI osimertinib, providing a model for identification of secondary mutations that confer resistance.

For this initial experiment, we designed three different pegRNAs programming the T to A base change at the first base of residue 797 in *EGFR* (Table S1). We performed an arrayed experiment in which we transiently co-transfected plasmids expressing each of these pegRNAs and the PE2 prime editor[22] into wild-type PC-9 cells. Two days later, the cells were treated with osimertinib, and, 24 days after drug treatment, cells were harvested and the *EGFR* locus amplified and sequenced. We observed a 16.8 to 25.3% frequency of T to A edits from reads overlapping the *EGFR* locus across the three pegRNAs tested in contrast with a 0.31% frequency of T to A edits in the wild-type, untransfected control, presumably sequencing errors (Figure 1B). As PC-9 cells harbor 8–10 copies of *EGFR*,[35] we estimate that, on average, resistant cells had 1–2 mutated copies of *EGFR* after selection. This experiment confirmed the ability for three different pegRNAs, each encoding the same T to A mutation to (1) successfully program this mutation at a low but appreciable frequency, sufficient for subsequent selection; and (2) confer resistance to osimertinib.

Next, we sought to develop an experimental screening framework in which cells stably express pegRNAs, such that pegRNA identities can be read out by directly sequencing integrated pegRNAs in place of sequencing the edited locus. We modified the LentiGuide-Puro-P2A-EGFP vector[36] to allow for cloning of pegRNAs (Figure S1). In addition to this lentiviral integration strategy, we also employed the engineered pegRNA (epegRNA) construct design, which incorporates an RNA stabilizing motif.[37] We also switched from using the PE2 prime editor to using the PEmax prime editor to enable higher rates of editing.[38] We cloned the same three pegRNAs that we used in the transient transfection experiment (Figure 1B) into this modified lentiviral vector and individually transduced these lentiviral epegRNA constructs into wild-type PC-9 cells. At 24 days after osimertinib treatment, cells were harvested and the *EGFR* locus was amplified and sequenced. Two of the three virally delivered epegRNAs successfully edited the EGFR locus at appreciable frequencies and conferred resistance to osimertinib treatment (Figures 1B–1D). This experiment confirmed our ability to perform prime editing experiments using integrated epegRNAs but also highlighted a key challenge, which is that some guides may fail to edit at appreciable frequencies. For all future experiments, we designed up to four epegRNAs per intended mutation.

Multiplex prime editing resolves well characterized resistance mutations in *BRAF*, *KRAS*, *EGFR*, *RIT1*, *MET*, and *PIK3CA*

Encouraged by our ability to prime edit and select for cells harboring the EGFR C797S T>A mutation, we next performed a pilot screen to assess our ability to install mutations in multiple genes for drug resistance in a single pooled experiment and to then detect these via guide sequencing (Figures 2A and 2B). We engineered *MLH1*ko-PEmax-PC-9, a PC-9 cell line with an *MLH1* knockout, and integrated PEmax to increase the prime editing rate[38] (Figure S2). We then designed a library of 121 epegRNAs (Table S3; Table S4) programming 35 mutations in six oncogenes (*EGFR*, *KRAS*, *PIK3CA*, *RIT1*, *BRAF*, and *MET*; Figure 2A). Nearly all of these mutations have previously been hypothesized to confer resistance to osimertinib, except for the EGFR T790M mutation, which was included as a control as it is a known osimertinib-sensitive mutation.[32] A pilot screen showed that sequencing the genomic loci and epegRNAs targeting those loci showed similar changes in frequency (Figure S3), confirming our ability to sequence pegRNAs as a proxy for edited loci. We transduced *MLH1*ko-PEmax-PC-9 cells with this epegRNA library. After selecting for cells containing an integrated epegRNA with puromycin, we treated cells with osimertinib. Cells were treated with three different concentrations of osimertinib (100, 300, and 500 nM) and harvested at 3, 7, 10, 14, 17, 21, and 24 days after drug treatment to profile the rate at which variants were proliferating throughout the time course. Replicates were well correlated in this screen across all time points (Pearson's r = 0.73, 0.76, and 0.77 for 100, 300, and 500 nM screens, respectively; Figures S4B–S4D). From this screen, we identified 18 statistically significant drug-resistant variants (log2 fold change [log2FC] > 0, $p < 0.05$, unpaired two-sided *t* test between variants and EGFR T790M at days 14–24), including BRAF V600E, EGFR S768I, EGFR G796D, EGFR L792H, EGFR G796R, EGFR C797S, EGFR C797G, KRAS G12C, KRAS G12S, KRAS G12V, KRAS G12D, KRAS Q61L, RIT1 M90I, PIK3CA R88Q, PIK3CA E453K, a MET 1010 splice site G>T variant, a MET 1010 splice site T>C variant, and MET Y1003* early stop codon variant, which is known to cause *MET* exon 14 skipping[39] (Figures 2C and S4A; Tables S6, S7, and S8). Some of the candidate osimertinib resistance variants were not significantly enriched, but we cannot distinguish between the possibility that these programmed prime edits were unsuccessful vs. a subset of these variants not being *bona fide* resistance mutations. As expected, cells with the EGFR T790M mutation did not proliferate in the presence of osimertinib (Table S8). We concluded that a 300 nM osimertinib treatment and a time point of 10–14 days, which corresponds roughly to 7–10 doublings (Figure S5), resulted in high signal in this screen (strictly standardized mean difference between EGFR C797S T>A and EGFR T790M = 14.1–14.4). The results of this pilot screen demonstrated that this experimental framework is capable of identifying mutations that confer resistance to TKIs (Figure 2C).

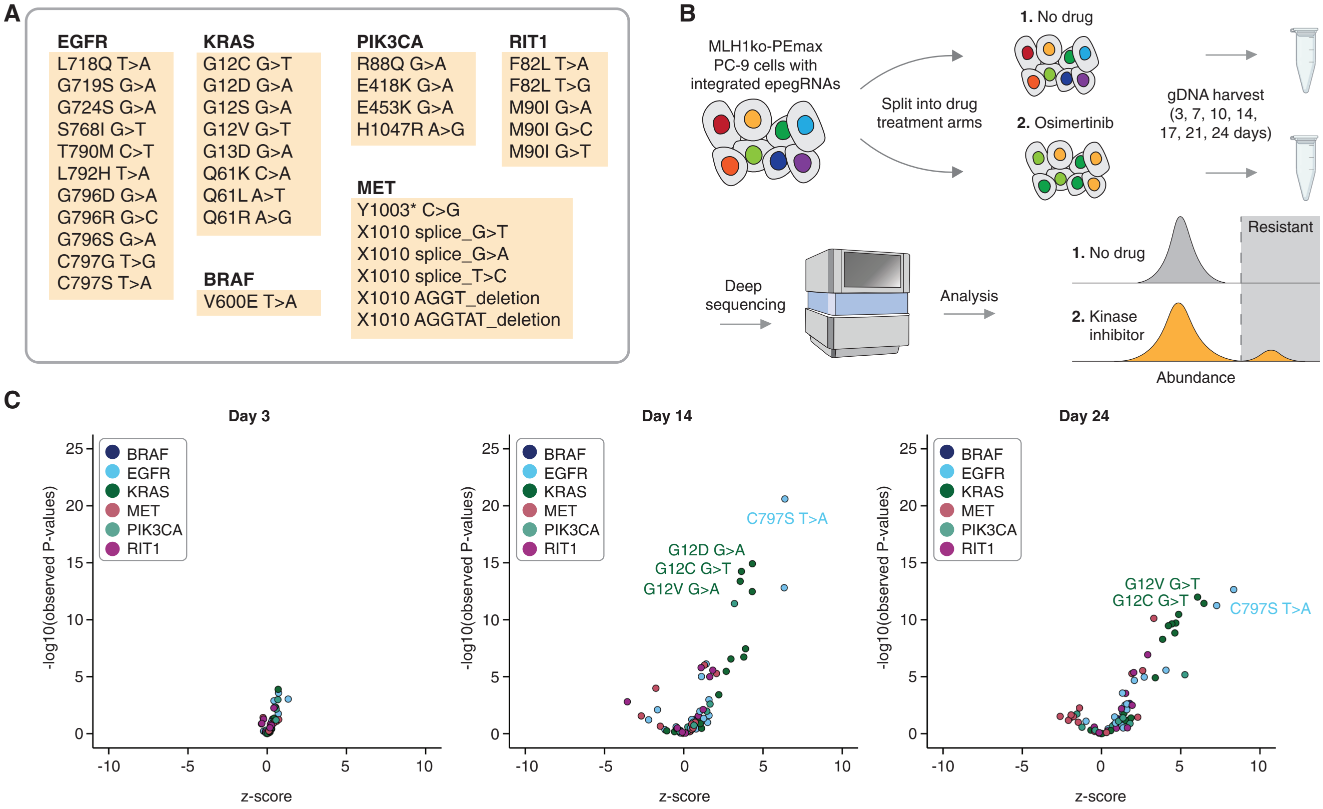

Figure 2. Proof-of-concept multiplexed prime editing of EGFR, KRAS, BRAF, PIK3CA, MET, and RIT1

(A) The 35 edits programmed by the 121 epegRNA pooled library.

(B) A pool of 121 epegRNAs was lentivirally integrated into *MLH1*ko-PEmax PC-9 cells and split into a no drug and an osimertinib drug treatment arm for 24 days. Genomic DNA was harvested, and integrated epegRNAs were amplified and sequenced.

(C) Volcano plots showing the *Z* scores and *p* values (unpaired two-sided *t* test, Bonferroni corrected) for epegRNAs in the 121 epegRNA pooled library screen at days 3, 14, and 24.

The results from this screen also highlighted the differential resistance phenotypes of these 18 resistant variants. EGFR C797S and KRAS G12 variants are the most well documented and well characterized osimertinib resistance mutations, and we observed that these variants vastly outcompete all the other identified resistant variants (Figure 2C). This differential resistance phenotype indicates that this screening framework can possibly rank variants by their degree of resistance by quantifying the relative fitness of a variant within a pool of cells of many variants, similar to growth-based deep mutational scans[40] and possibly even resembling clonal competition that happens during tumor growth.

Large-scale testing of drug resistance mutations with three inhibitors across seven oncogenes

Osimertinib is the current standard therapy for non-small cell lung cancer patients harboring an EGFR T790M mutation. However, resistance to osimertinib typically develops, on average, within 10 months of treatment due to histological transformation or the acquisition of oncogene amplifications or other resistance mutations.[41] Because of this, newer fourth-generation TKIs have been developed to treat osimertinib-resistant non-small cell lung cancers. Two of these newer inhibitors include sunvozertinib and CH7233163. Sunvozertinib, which is approved in China and recently received Breakthrough Therapy designation from the US Food and Drug Administration (FDA) for Exon20+ non-small cell lung cancer, irreversibly and covalently binds EGFR at the C797 residue and is able to treat tumors that have an *EGFR* exon 20 insertion that renders these cells resistant to osimertinib.[42] CH7233163 is a next-generation *EGFR* inhibitor that is a non-covalent, competitive binder of the ATP-binding pocket of EGFR[43] and is currently in preclinical development for treatment of non-small cell lung cancers that are resistant to third-generation inhibitors.[44] Mechanistically, osimertinib and sunvozertinib are similar in that they irreversibly and covalently bind EGFR C797, and CH7233163 differs in that it is a non-covalent, competitive binder of the ATP-binding pocket.

Recognizing the unique potential of prime-SGE to scale without the need to sequence each targeted locus, we next asked whether we could apply it to saturate exons and splice site regions of various known oncogenes to screen thousands of single nucleotide variants for resistance to multiple different TKIs in a single experiment. We designed 3,825 epegRNAs programming 1,220 single nucleotide mutations, both missense and synonymous, or deletions in seven different genes (*EGFR*,

CellPress
OPEN ACCESS

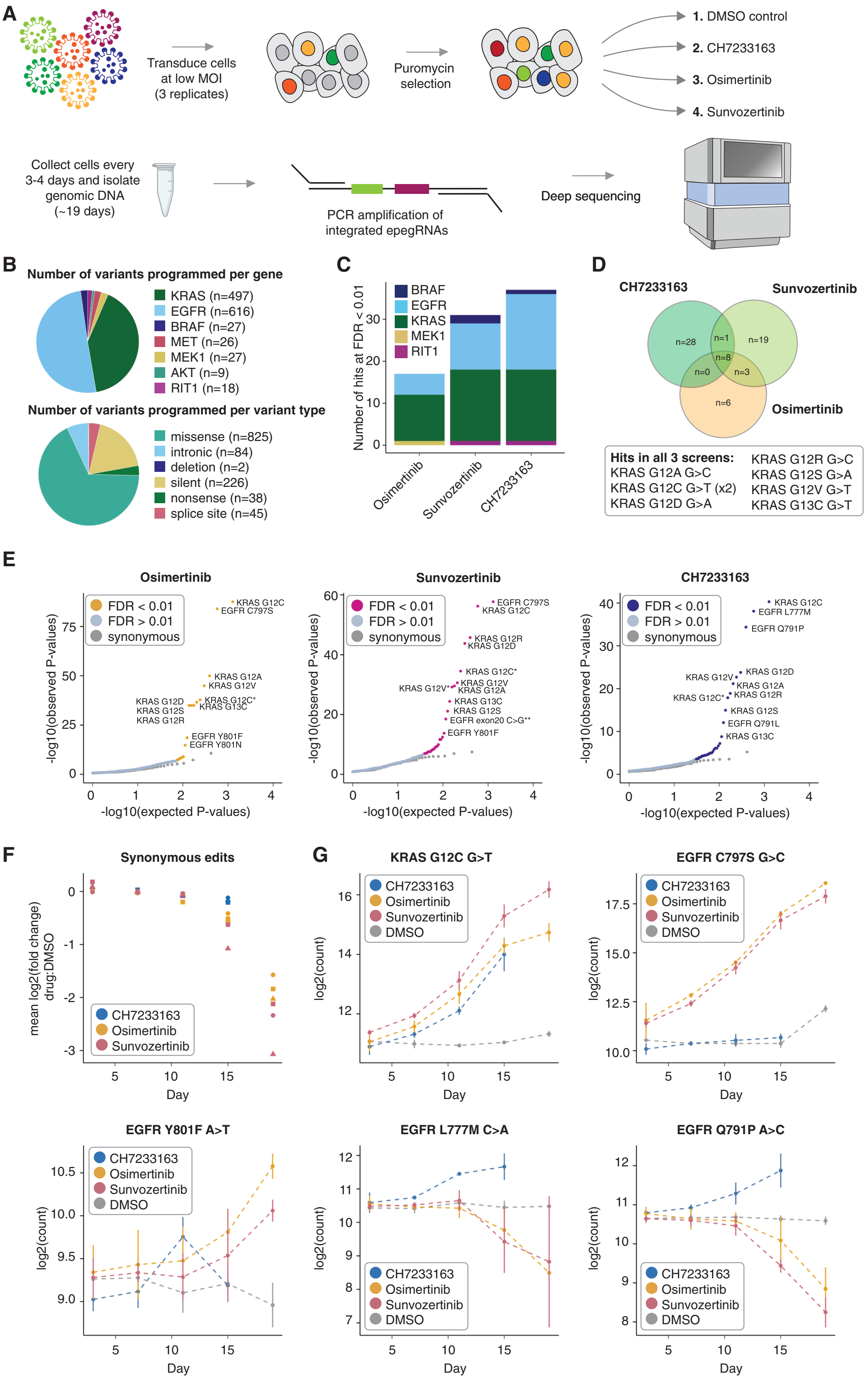

(legend on next page)

KRAS, *MET*, *RIT1*, *BRAF*, *MEK1*, and *AKT*; Figures 3A and 3B; Tables S7, S9, S10, and S11). In designing these epegRNAs, we also included a randomized eight nucleotide barcode directly 3′ of the epegRNA terminator sequence (Figure S1E). The rationale for this barcode was to understand whether resistant cells were clonally derived or whether independent introductions of the same mutation recurrently resulted in resistance.

After generating lentivirus, cells were transduced in triplicate at >2,000× coverage into *MLH1*ko-PEmax-PC-9 cells at a multiplicity of infection (MOI) of 0.35 (Figure S6). After puromycin selection, cells were subjected to one of four treatment conditions: DMSO, CH7233163, osimertinib, or sunvozertinib (Figure 3A). Cells were harvested at days 3, 7, 11, 15, and 19, and genomically integrated epegRNAs were amplified and sequenced (Figure 3A). epegRNA read counts were correlated across replicates, time points, and drug treatments (Figure S7).

We employed DESeq2[45] to identify hits from this large screen by identifying variants that are differentially abundant between the DMSO control and the three different drug treatments (CH7233163, osimertinib, and sunvozertinib) over the time course of 19 days. A likelihood ratio test (LRT) was performed between a reduced model that includes the variable time and a full model that includes the variables time, drug treatment, and the interaction of drug treatment and time. Synonymous variants were used as controls for false discovery rate (FDR) testing (STAR Methods). From the results of this LRT, we identified 17 differentially abundant variants in the osimertinib screen, 31 differentially abundant variants in the sunvozertinib screen, and 37 differentially abundant variants in the CH7233163 screen (FDR < 0.01, log2FC > 0; Figures 3C–3G and S8G; Tables S12, S13, and S14).

3,825 epegRNA screens identify drug resistance mutations in EGFR and KRAS *in vitro* and in an *in vivo* mouse xenograft model

There were seven unique resistant variants that were hits in all three screens, and all seven of these are variants at the 12th and 13th residues of the *KRAS* oncogene (Figure 3D). The *KRAS* gene, which is part of the RAS family, is one of the most frequently mutated genes in cancer. Mutations in *KRAS* are a major driver of lung cancers,[46] particularly the *KRAS* G12C mutation. *KRAS*, until the recent approval of sotorasib,[47] was considered to be undruggable due to four decades of failed drug-discovery attempts to target this oncogene.[46] Mutations at the 12th and 13th residues in the phosphate-binding loop of *KRAS* are known oncogenic drivers as these mutations directly impair the ability of KRAS to hydrolyze guanosine triphosphate (GTP). This causes the protein to remain in an active GTP-bound state, leading to continued cell growth and the development of cancer. Mutations in *KRAS* do not cause drug resistance by directly inhibiting a drug from binding its target site but rather by re-activating oncogenic signaling pathways that lead to constitutive signaling and cell growth. All programmed G12 missense mutations were hits in all three screens ($p < 2.27 \times 10^{-7}$, LRT), but only a single G13 (G13C) mutation was a hit in all three screens ($p < 1.58 \times 10^{-9}$, LRT). KRAS G13D is a known oncogenic mutation,[48] suggesting that the KRAS G13D variant may not have been successfully installed into the genomic DNA via prime editing in this screen. To test this hypothesis, we designed new epegRNAs using a more advanced design algorithm, PRIDICT 2.0,[49] and tested them head to head with our original epegRNAs for KRAS G13D along with other EGFR and PIK3CA variants. We found that PRIDICT 2.0-designed pegRNAs did not lead to a higher editing rate of G13D, suggesting that G13D is a difficult mutation to make by prime editing (Figure S9).

Another variant that we would expect to give rise to resistant cells in the osimertinib and sunvozertinib screens, but not in the CH7233163 screen, is EGFR C797S, and this is exactly what we observe (Figure 3G). EGFR C797S was a strong hit in the osimertinib and sunvozertinib screens ($p = 1.14 \times 10^{-84}$ and $p = 2.28 \times 10^{-55}$, respectively; LRT), whereas it was not a hit in the CH7233163 screen ($p = 0.34$, LRT) (Figure 3G). CH7233163 overcomes the EGFR L858R/T790M/C797S triple mutation,[43] and we observe that EGFR C797S is not a hit when cells are treated with CH7233163, suggesting that EGFR C797S does not confer resistance to cells against this inhibitor. CH7233163 differs from both osimertinib and sunvozertinib in that it is not a covalent binder to the EGFR ATP-binding site but, rather, is a non-covalent ATP-competitive inhibitor of EGFR. This difference in binding mechanism could explain the differential resistance profiles we observe in cells treated with

Figure 3. Drug resistance screen of 1,220 mutations against three different EGFR inhibitors

(A) A pool of 3,825 epegRNAs was lentivirally transduced into *MLH1*ko-PEmax PC-9 cells at a low MOI. Cells were selected with puromycin and split into one of four treatment arms (DMSO, CH7233163, osimertinib, and sunvozertinib). Cells were harvested every 3–4 days for over 19 days, and integrated epegRNAs were amplified and sequenced from genomic DNA.

(B) Top: pie chart showing the number of variants programmed for each of seven genes. Bottom: pie chart showing the number of variants programmed for each type of mutation.

(C) Stacked bar plot showing the number of resistant hits in each screen, colored by gene. Hits are variants that are at an FDR < 0.01 and a log2FC > 0 by DESeq2 differential epegRNA abundance analysis.

(D) Venn diagram showing the overlap of resistant hits between the three different drug treatment screens.

(E) Quantile-quantile plots showing the distribution of expected versus observed *p* values for the three different drug screens. Significance was calculated using biological replicate samples (i.e., independent lentiviral transductions; $n = 3$). Each point represents a unique genetic variant encoded by 1–4 epegRNAs. Variants with an asterisk (*) after the label indicate variants that were programmed with an additional synonymous mutation in the neighboring codon. Variants with a double asterisk (**) after the label indicates variants that are splice junction variants.

(F) Mean of the log2 fold change of synonymous variants in the three drug screens when compared to the DMSO control. Shapes represent three biological replicates.

(G) Individual variant trends in drug-treated cells and DMSO-treated cells. The *y* axis represents normalized log2(read counts) of variant pegRNAs. Points represent the mean of three replicates, and error bars represent one standard deviation from the mean of the three replicates.

CellPress
OPEN ACCESS

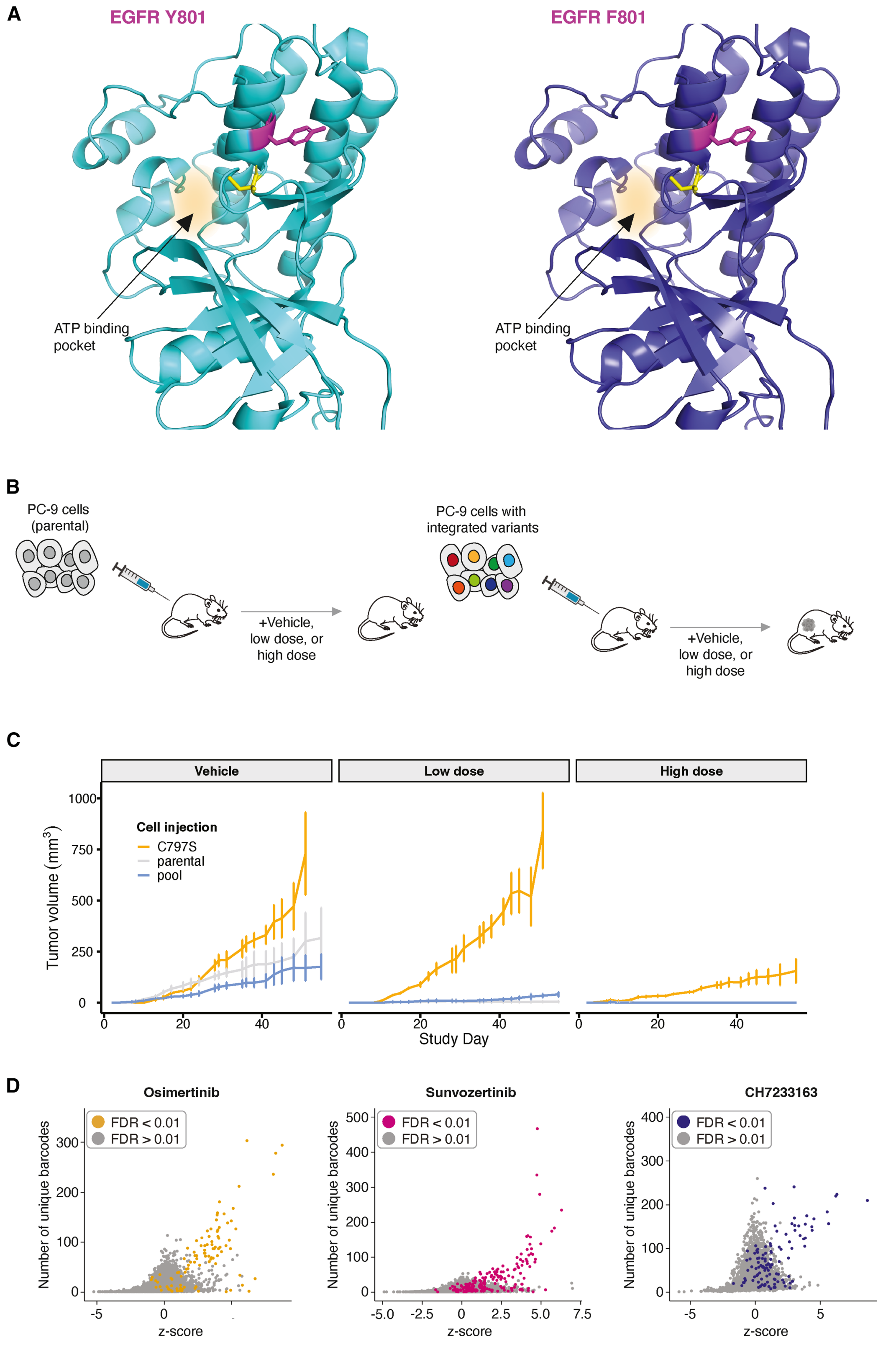

(legend on next page)

this inhibitor, such as the lack of EGFR C797S as an identified resistance mutation. Because EGFR C797S is well edited in this and previous experiments, and because cells in the different drug arms came from the same starting pool of edited cells, it is unlikely to be a false negative in the CH7233163 treatment arm.

In addition to identifying well-characterized resistance mutations, we also identified numerous missense mutations that confer resistance to TKI treatment that are less well characterized. Examples of such mutations include EGFR Q791P (Figure 3G) and EGFR Q791L, which were both hits in the CH7233163 screen ($p = 4.35 \times 10^{-35}$ and $p = 8.44 \times 10^{-13}$, respectively). EGFR Q791 missense mutations are not extensively documented as being drivers of TKI resistance, and documented cases of EGFR Q791 in the context of lung cancer, while present, are sparse.[50,51] Mutations in Q791 are predicted to reduce the binding affinity of EGFR to osimertinib.[52] EGFR Y801 is another example of a residue that, when mutated, has been identified in single cases of lung cancer,[53] malignant peritoneal mesothelioma,[54] gastric carcinoma,[55] and two cases of squamous cell carcinoma,[56,57] but it is not known for certain whether a mutation at this residue is a primary driver of resistance to TKIs. EGFR Y801 is a well-conserved residue[54] and lies within the activation loop of EGFR. EGFR Y801F (Figures 3G and 4A) and Y801N are hits in both the osimertinib and sunvozertinib screens ($p < 5.96 \times 10^{-8}$; Tables S13 and S14) but not in the CH7233163 screen (Tables S12, S13, and S14). Although our screening framework is not able to conclusively identify non-resistant variants due to the fact that we cannot be certain that prime edits were made, the fact that these EGFR Y801 variants are resistant in two of the three screens is highly suggestive that EGFR Y801 missense variants are successfully being installed by the prime editing machinery and that they are likely sensitive to CH7233163. Furthermore, the difference in mechanism of EGFR binding between the two covalent inhibitors (osimertinib and sunvozertinib) and the non-covalent inhibitor (CH7233163) plausibly underlies the difference in resistance mutations we observe between these two classes of inhibitors, such as is the case for EGFR C797S. The ability of our screening method to identify rare resistance mutations makes it useful for identifying unknown resistance mutations that have not yet been documented in cancer-sequencing databases.

To demonstrate prime-SGE in an *in vivo* context, we assessed the ability of prime-edited cells to form tumors while being treated with osimertinib in a mouse xenograft model (Figure 4B). MLH1ko-PEmax-PC-9 cells harboring wild-type EGFR (parental cells), EGFR C797S cells, or a pool of eight variants (KRAS G12C, EGFR Y801F, EGFR Y801*, EGFR L777M, EGFR Q791L, EGFR Q791*, EGFR Q791P, and EGFR T790M; Table S17) were subcutaneously injected into immunocompromised mice. While the parental cells under osimertinib treatment consistently failed to form tumors in the injected mice, as expected, the C797S cells, for all dilutions down to 1:1,000, under osimertinib treatment, formed tumors within 2 weeks of injection ($n = 18$ mice, six per condition) (Figure S10). Notably, the pool of eight variants also resulted in tumor formation, although these tumors were much smaller in size than those formed with EGFR C797S cells (Figure 4C). Sequencing results from this pool showed that the pool was dominated by the KRAS G12C variant, which suggests that this variant has a strong resistant phenotype, potentially dominating other resistant variants in the pool (Figure 4C; Table S18). These findings suggest that resistant mutations installed by prime editing lead to tumor resistance *in vivo*, while unedited cells do not.

Barcoded epegRNAs elucidate clonality of resistant cell populations and their growth trajectories

Two features of prime-SGE have the potential to yield unique insights into the emergence and growth behavior of resistant cells. The first of these is the inclusion of a pegRNA-specific barcode that is directly 3′ of the epegRNA terminator sequence (Figure S1E). The placement of this barcode does not affect epegRNA binding to its target site, as the barcode is not transcribed, but it allows us to amplify and sequence the barcode identity alongside the epegRNA from genomic DNA. The inclusion of this barcode in the epegRNA design allows us to determine whether resistant cells arose from few or many editing events. To leverage this feature, we first calculated a Z score for each variant to determine its enrichment in the drug-treatment conditions vs. control. Next, we examined the relationship between Z scores and the number of underlying barcodes (Figure 4D). This analysis shows that many resistant variants (as identified by DESeq2) are characterized by a high number of unique barcodes (Figures 4D and S8A–S8F). This suggests that, for the most part, resistant cells arose from multiple independent editing events that introduced the underlying resistance mutation. As DESeq2 did not leverage these guide embedded barcodes, this provides orthogonal support for the classification of many of these hits as resistant. Finally, although this warrants further investigation, this result also suggests that the low efficiency of prime editing is highly target specific; i.e., some targets edit well, whereas others are rarely or not at all edited.

Figure 4. Alpha fold structure prediction, extension of prime-SGE to an *in vivo* mouse xenograft model, and barcode analysis in the PC-9 large-scale screen

(A) Left: AlphaFold-predicted structure of EGFR Y801. Right: AlphaFold-predicted structure of EGFR F801. Residue 801 is in magenta, C797 residue is in yellow, and the ATP-binding pocket is highlighted.

(B) Pools of parental or prime-edited PC-9 cells (C797S or a pool of variants) were implanted subcutaneously into mouse flanks. Mice were treated with osimertinib or vehicle control. Tumor growth was measured 3× weekly.

(C) Visualization of tumor volume over time in mice injected with wild-type PC-9 cells (parental; gray curve), PC-9 harboring the *EGFR* C797S variant (C797S; orange curve), or a pool of PC-9 cells harboring eight *KRAS* and *EGFR* variants. Plots are faceted by treatment group, including vehicle (DMSO control), low-dose osimertinib (5 mg/kg), and high-dose osimertinib (20 mg/kg). The plotted lines represent the mean of tumor volumes measured, and error bars represent one standard deviation from the mean.

(D) Relationship between the Z score and the number of unique barcodes recovered for a given epegRNA. Plots shown include data from two time points (day 15 and day 19) and three replicates. Each point represents a unique genetic variant encoded by 1–4 epegRNAs at a single time point and a single replicate. Points are colored by FDR.

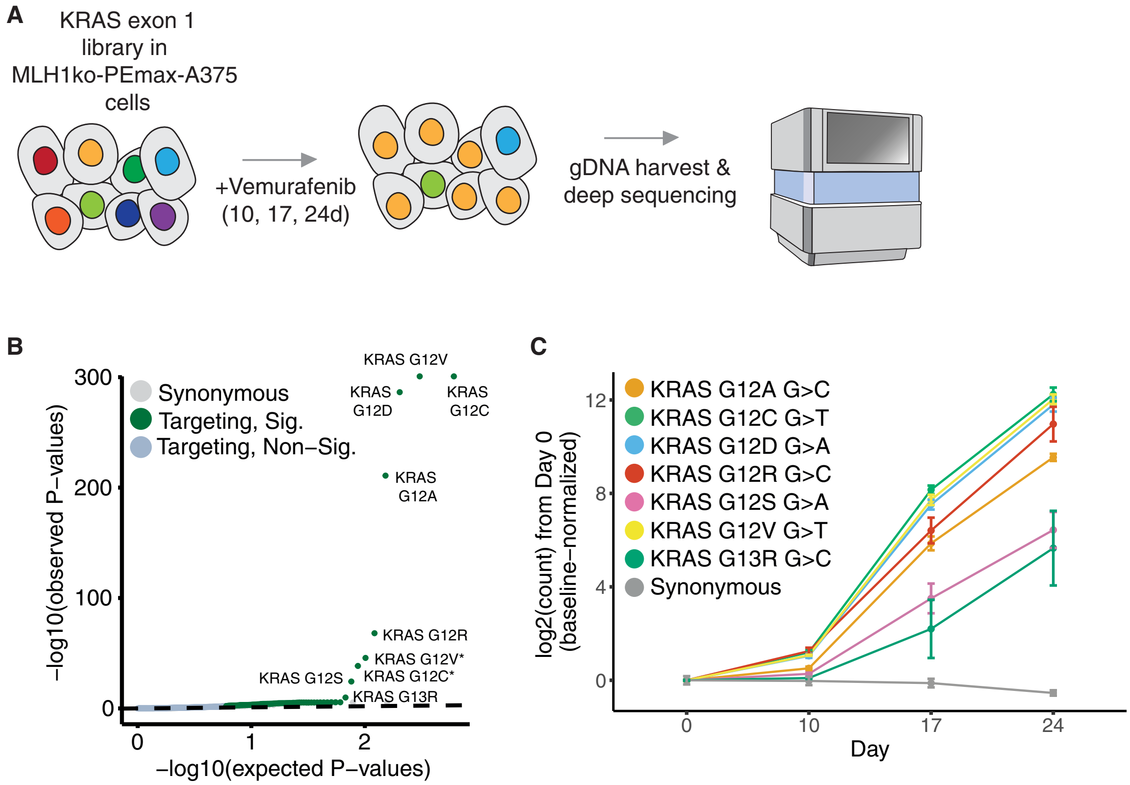

Figure 5. Resistance screening in an additional cell line and drug context

(A) A library of 1,228 epegRNAs programming KRAS exon 1 variants were transduced at low MOI into MLH1ko-PEmax-A375 cells. Cells were selected with puromycin and split into one of two treatment arms (DMSO and vemurafenib). Cells were harvested at 10, 17, and 24 days, and integrated epegRNAs were amplified and sequenced from genomic DNA.

(B) Quantile-quantile plot showing the distribution of expected versus observed *p* values for the KRAS exon1 screen in MLH1ko-PEmax-A375 cells selected with vemurafenib. Each point represents a unique genetic variant encoded by 1–4 epegRNAs. Variants with an asterisk (*) after the label indicate variants that were programmed with an additional synonymous mutation in the neighboring codon.

(C) Abundance trends for the top seven hits in the vemurafenib screen in A-375 cells across the time course. Points represent the mean of three replicates, error bars represent one standard deviation from the mean of three replicates.

A second feature of prime-SGE experimental design is that it allows us to observe the trajectory of resistant cells at high resolution via multiple harvests over the 19 day time course (Figures 3F and 3G). Consistent trajectories over the time course add further confidence to hits; if a variant is increasing in abundance over the time course of 19 days in a drug-treatment condition as compared to a DMSO control, we can be more certain it is a *bona fide* resistance mutation. This is useful in a prime editing screen, as the presence of a pegRNA does not guarantee the presence of the programmed mutation. Particularly when identifying potential novel drug-resistance mutations, such as EGFR Y801F, observing a consistent increase in the frequency of this variant over the time course gives further support to the identification of this missense variant as a statistically significant hit in this screen (Figure 3G).

Prime-SGE identifies resistant variants across different contexts

To demonstrate that prime-SGE is robust and applicable beyond PC-9 cells and EGFR inhibitors, we performed a prime-SGE screen targeting all possible single-nucleotide variants in KRAS exon 1 in A-375 melanoma cells to identify variants that confer resistance to a BRAF inhibitor. Using a library of 1,228 epegRNAs programming 498 KRAS exon 1 variants (Table S19), cells were transduced at low MOI into MLH1ko-PEmax-A375 cells and treated with vemurafenib, a BRAF inhibitor (Figures 5A and S11; STAR Methods). Vemurafenib specifically inhibits the activity of BRAF V600E, which is a common oncogenic driver mediated by aberrant signaling through the MAPK/ERK pathway. Cells were harvested at 10, 17, and 24 days following the start of vemurafenib treatment. Following sequencing of integrated epegRNAs, we performed differential abundance analysis using DESeq2 to identify resistant variants. In total, we identified 87 resistant variants in this screen (DESeq2 Wald test, T3 vs T0 contrast, one-sided, Benjamini–Hochberg FDR < 0.05; Figure 5B; Table S20). Vemurafenib resistance was strongly mediated by KRAS G12 and G13 variants, which are known to be tumorigenic and resistant to kinase inhibitors. The top four resistant variants in this screen were KRAS G12A, G12C, G12D, and G12V, and their resistant growth phenotype is in sharp contrast to that of synonymous variants in the screen (Figure 5C). Notably, G13D, a known resistance mutation, was not a hit in this screen either, confirming the difficulty in making this prime edit. These findings confirm the versatility of

prime-SGE as a screening platform across different cellular contexts and reinforce the critical contribution of KRAS G12 (Figure 5C) mutations to resistance phenotypes in melanoma.

DISCUSSION

Here, we describe prime-SGE, a multiplex, prime editing-based screening framework to identify drug resistant variants. A key feature of prime-SGE is that it installs genomic edits via prime editing, which uses a single pegRNA to both encode the target site and the programmed edit. The identity of the pegRNA, and its unique barcode, is read out by sequencing amplified pegRNAs from genomic DNA, which allows for increased scaling of this method to many variants in many exons in many genes in a single screen. Furthermore, individually barcoded epegRNAs enable discrimination and tracking of independently originating editing events, something that is not possible with current SGE methods. In applying this method to several oncogenes and several TKIs in a lung adenocarcinoma and melanoma cell line, we were able to resolve well-characterized resistance mutations and oncogenic driver mutations, such as EGFR C797S and KRAS G12 missense variants, as well as less well-characterized resistance mutations, such as EGFR Q791 and Y801 missense variants.[53–57] We note that EGFR Y801 mutations were not identified as resistant to osimertinib in a previous prime editing screen and suspect that this screen suffered from lack of sensitivity due to the low abundance of informative sequencing reads.[25,52,58] We also observed differential resistance phenotypes between covalent and non-covalent binders of EGFR, providing a means to directly compare the resistance behavior of programmed variants across different classes of inhibitors. Looking forward, prime-SGE holds the potential to screen large numbers of genetic variants, at locations throughout the genome, for resistance to any number of inhibitors.

While this work was underway, several related studies were published that implement multiplex prime editing screens in ways that are complementary to prime-SGE. A first set relies on sequencing the endogenous locus or loci to quantify the abundance of specific edits.[24–26] However, this undercuts a key advantage of multiplex prime editing, as targeting of multiple exons or genes would require each to be independently amplified and sequenced. A second set uses prime editing to program edits to multiple loci but then either sequence an accompanying sensor cassette[28,29] or, like prime-SGE, sequence the pegRNA cassette.[27] However, these strategies are implemented as loss-of-function screens and do not fully solve the issue of false negatives resulting from low editing rates. The specific advances introduced here are (1) deploying a positive-selection framework, which inherently yields higher confidence in hits than loss-of-function designs; and (2) applying multiplex prime editing to systematically identify, validate, and characterize drug resistance mutations, previously only accessible through low-throughput, labor-intensive approaches. In our system, drug resistant clones can expand only if they harbor a programmed resistance-conferring edit, sharply reducing false positives. Given that prime editing efficiencies are often in the single-digit range, this positive-selection architecture is arguably essential for obtaining reliable, interpretable screening results.

The prime editing field is advancing at a rapid pace, and we were able to incorporate numerous improvements that were reported while prime-SGE was under development. These include knocking out *MLH1*, which has been shown to improve editing efficiency,[38] utilizing PEmax, a further engineered nCas9-RT, and incorporating an improved enhanced pegRNA structural design that includes a 3′ RNA stabilizing motif.[37] With further improvements in editing efficiency, we expect the potential of prime-SGE to grow. With continued improvements, it is also plausible to use this screening framework as a way to identify drug sensitive variants; to assay drug resistance behavior to combination therapies; to envision prime editing-based screens being used for the large-scale identification of loss-of-function mutations, as has been recently demonstrated[25–27]; and other functional screening applications. Looking forward, we also envision prime-SGE and related methods being applied in increasingly complex cell types and model systems. Particularly if the efficiency issues can be overcome, prime-SGE and related methods have the potential to greatly accelerate the functional annotation of the extensive list of coding and non-coding variants that underlie risk for both Mendelian and complex human diseases.

Limitations of the study

Although prime-SGE is able to resolve many drug resistance mutations, a major caveat of this screening framework is that it is unable to unequivocally identify all resistant variants. Despite the considerable effort that we put into designing effective pegRNAs,[37,59,60] for any given programmed edit, there remains a high degree of uncertainty that an edit occurred. For example, mutations at KRAS G13 are known oncogenic drivers. However, in our four drug screens, we only identified a single KRAS G13 missense mutation as a hit (KRAS G13C), despite the fact that five other epegRNAs programming G13 missense mutations were present in the library, one of which is a known oncogenic mutation (KRAS G13D).[48] This suggests that at least the G13D missense variant was not successfully edited into the genomic DNA in either of our cell lines. This false negative result for KRAS G13D surely extends to other variants and suggests that we identified fewer drug resistant variants than the true number. From screening 1,220 mutations in PC-9 cells, we identified 17, 31, and 37 drug resistant hits in the osimertinib, sunvozertinib, and CH7233163 screens, respectively. This represents roughly a 2.3% hit rate. We screened 498 mutations in A-375 cells and identified 87 drug resistant hits, representing a 17.5% hit rate in this screen. Although it is challenging to determine the false negative rate of these screens since only a handful of resistance mutations are confidently known, we estimated, on average, a 40% false negative rate by assessing how many known osimertinib resistance mutations were not identified (Table S15). Looking forward, further improvements to prime editing efficiency and/or pegRNA design should mitigate this issue.

RESOURCE AVAILABILITY

Lead contact

Further information and requests for resources and reagents should be directed to and will be fulfilled by the lead contact, Lea M. Starita (lstarita@uw.edu).

Materials availability

Materials used in this study can be found in the key resources table as well as in the STAR Methods section of this paper. Additional materials are available from the lead contact upon request.

Data and code availability

- Data have been deposited into FigShare and are publicly available as of the date of publication at Figshare: https://figshare.com/projects/A_multiplex_prime_editing_framework_for_identifying_drug_resistance_variants_at_scale/247232.
- Data from this paper are also publicly available and accessible via the following website: https://krishna.gs.washington.edu/content/members/multiplex_PE_screening/public/.
- All original code has been deposited into FigShare and is publicly available at Figshare: https://figshare.com/projects/A_multiplex_prime_editing_framework_for_identifying_drug_resistance_variants_at_scale/247232 as of the date of publication.

ACKNOWLEDGMENTS

We are grateful to members of the Starita, Berger, and Shendure labs for comments, suggestions, and discussions on this work. We are especially grateful for Junhong Choi's feedback during the initial proof-of-concept experiments and for discussions with Cole Trapnell regarding how to properly analyze these data. We are also grateful to the Shendure lab gene regulation subgroup for technical advice and deep discussions regarding the development of the prime editing screening method. We thank Elizabeth Cromwell and the Fred Hutch Preclinical Modeling Core for advice and technical assistance with the mouse xenograft studies. PC-9 cells were a gift from the M. Meyerson lab at the Broad Institute. pSpCas9(BB)-2A-Puro (PX459) V2.0 (Addgene #62988) was a kind gift from the F. Zhang lab at the Massachusetts Institute of Technology. LentiGuide-Puro-P2A-EGFP (Addgene plasmid #137729), which was used as the base vector to create the Lenti-epeg-Puro-P2A-EGFP, was a kind gift from the F. Wermeling lab at the Karolinska institute. The pCMV-PE2 plasmid (Addgene #132775), pU6-pegRNA-GG-acceptor (Addgene plasmid #132777), and the pCMV-PEmax plasmid (Addgene #174820) were a kind gift from the D. Liu lab at Harvard University. This work was supported by the NIH NHGRI grant 5RM1HG010461 to L.M.S and J.S. C.C.S. was supported by the NIGMS (T32GM136534) and NHLBI (F31HL168982). P.P. was supported by a National Science Foundation (NSF) graduate research fellowship. T.A.M. was supported by a Banting Postdoctoral Fellowship from the Natural Sciences and Engineering Research Council of Canada (NSERC). J.-B.L. was a Fellow of the Damon Runyon Cancer Research Foundation (DRG-2435-21). D.C. was supported by the NHGRI (F32HG011817). J.S. is an investigator of the Howard Hughes Medical Institute. A.H.B. was supported in part by the American Cancer Society Research Scholar Grant RSG-21-090-01 and NIH NCI grant R37CA252050.

AUTHOR CONTRIBUTIONS

Conceptualization, F.M.C.A., A.H.B., J.S., and L.M.S.; investigation, F.M.C.A.; data curation, F.M.C.A., C.C.S., R.M.D., N.T.S., M.C.R., and P.P.; formal analysis, F.M.C.A.; visualization, F.M.C.A.; resources, J.S. and L.M.S.; supervision, A.H.B., J.S., and L.M.S.; writing – original draft, F.M.C.A.; writing – review & editing, F.M.C.A., C.C.S., R.M.D. N.T.S., M.C.R., B.M., P.P., T.A.M., J.-B.L., D.C., A.H.B., J.S., and L.M.S.; funding acquisition, J.S. and L.M.S.

DECLARATION OF INTERESTS

J.S. is a scientific advisory board member, consultant, and/or co-founder of Prime Medicine, Guardant Health, Camp4 Therapeutics, Phase Genomics, Adaptive Biotechnologies, Sixth Street Capital, Pacific Biosciences, Cellular Intelligence, and 10× Genomics.

STAR★METHODS

Detailed methods are provided in the online version of this paper and include the following:

- KEY RESOURCES TABLE
- EXPERIMENTAL MODEL AND STUDY PARTICIPANT DETAILS
  - Cell lines and culture
- METHOD DETAILS
  - Piggybac-PEmax vector cloning
  - *MLH1* knockout-PEmax cell line generation and validation in PC-9 cells
  - *MLH1* knockout-PEmax cell line generation and validation in A-375 cells
  - pegRNA selection and design
  - pegRNA cloning into transient and lentiviral vectors
  - Lentivirus generation
  - Lentivirus titration experiment in 3,825 epegRNA lentiviral pooled editing screens
  - Osimertinib dose titration curves
  - pegRNA transient transfection or lentiviral transduction into PC-9 cells
  - pegRNA lentiviral transduction into MLH1ko-PEmax-A375 cells
  - Drug treatments used in screens
  - Mouse xenograft cell injections and tumor growth data collection
  - Cell harvests and genomic DNA extraction for the 3,825 epegRNA lentiviral pooled editing screens
  - pegRNA amplicon amplification and sequencing
- QUANTIFICATION AND STATISTICAL ANALYSIS
  - Raw data processing and quality control filtering of sequencing data

SUPPLEMENTAL INFORMATION

Supplemental information can be found online at https://doi.org/10.1016/j.xgen.2026.101167.

Received: September 19, 2023
Revised: November 23, 2025
Accepted: January 26, 2026
Published: February 20, 2026

REFERENCES

1. Vasan, N., Baselga, J., and Hyman, D.M. (2019). A view on drug resistance in cancer. Nature *575*, 299–309.
2. Glickman, M.S., and Sawyers, C.L. (2012). Converting cancer therapies into cures: lessons from infectious diseases. Cell *148*, 1089–1098.
3. Konieczkowski, D.J., Johannessen, C.M., and Garraway, L.A. (2018). A Convergence-Based Framework for Cancer Drug Resistance. Cancer Cell *33*, 801–815.
4. Reita, D., Pabst, L., Pencreach, E., Guérin, E., Dano, L., Rimelen, V., Voegeli, A.-C., Vallat, L., Mascaux, C., and Beau-Faller, M. (2021). Molecular Mechanism of EGFR-TKI Resistance in EGFR-Mutated Non-Small Cell Lung Cancer: Application to Biological Diagnostic and Monitoring. Cancers *13*, 4926. https://doi.org/10.3390/cancers13194926.
5. Frampton, G.M., Fichtenholtz, A., Otto, G.A., Wang, K., Downing, S.R., He, J., Schnall-Levin, M., White, J., Sanford, E.M., An, P., et al. (2013). Development and validation of a clinical cancer genomic profiling test based on massively parallel DNA sequencing. Nat. Biotechnol. *31*, 1023–1031.
6. Zehir, A., Benayed, R., Shah, R.H., Syed, A., Middha, S., Kim, H.R., Srinivasan, P., Gao, J., Chakravarty, D., Devlin, S.M., et al. (2017). Mutational landscape of metastatic cancer revealed from prospective clinical sequencing of 10,000 patients. Nat. Med. *23*, 1004.
7. Garcia-Murillas, I., Schiavon, G., Weigelt, B., Ng, C., Hrebien, S., Cutts, R.J., Cheang, M., Osin, P., Nerurkar, A., Kozarewa, I., et al. (2015).

Mutation tracking in circulating tumor DNA predicts relapse in early breast cancer. Sci. Transl. Med. *7*, 302ra133.

8. Clark, T.A., Chung, J.H., Kennedy, M., Hughes, J.D., Chennagiri, N., Lieber, D.S., Fendler, B., Young, L., Zhao, M., Coyne, M., et al. (2018). Analytical Validation of a Hybrid Capture–Based Next-Generation Sequencing Clinical Assay for Genomic Profiling of Cell-Free Circulating Tumor DNA. J. Mol. Diagn. *20*, 686–702.
9. Overman, M.J., Modak, J., Kopetz, S., Murthy, R., Yao, J.C., Hicks, M.E., Abbruzzese, J.L., and Tam, A.L. (2013). Use of research biopsies in clinical trials: are risks and benefits adequately discussed? J. Clin. Oncol. *31*, 17–22.
10. Awad, M.M., Liu, S., Rybkin, I.I., Arbour, K.C., Dilly, J., Zhu, V.W., Johnson, M.L., Heist, R.S., Patil, T., Riely, G.J., et al. (2021). Acquired Resistance to KRASG12C Inhibition in Cancer. N. Engl. J. Med. *384*, 2382–2393.
11. Ramirez, M., Rajaram, S., Steininger, R.J., Osipchuk, D., Roth, M.A., Morinishi, L.S., Evans, L., Ji, W., Hsu, C.-H., Thurley, K., et al. (2016). Diverse drug-resistance mechanisms can emerge from drug-tolerant cancer persister cells. Nat. Commun. *7*, 10690.
12. Berger, A.H., Brooks, A.N., Wu, X., Shrestha, Y., Chouinard, C., Piccioni, F., Bagul, M., Kamburov, A., Imielinski, M., Hogstrom, L., et al. (2017). High-throughput Phenotyping of Lung Cancer Somatic Mutations. Cancer Cell *32*, 884.
13. Wagenaar, T.R., Ma, L., Roscoe, B., Park, S.M., Bolon, D.N., and Green, M.R. (2014). Resistance to vemurafenib resulting from a novel mutation in the BRAFV600E kinase domain. Pigment Cell Melanoma Res. *27*, 124–133.
14. Persky, N.S., Hernandez, D., Do Carmo, M., Brenan, L., Cohen, O., Kitajima, S., Nayar, U., Walker, A., Pantel, S., Lee, Y., et al. (2020). Defining the landscape of ATP-competitive inhibitor resistance residues in protein kinases. Nat. Struct. Mol. Biol. *27*, 92–104.
15. Findlay, G.M., Boyle, E.A., Hause, R.J., Klein, J.C., and Shendure, J. (2014). Saturation editing of genomic regions by multiplex homology-directed repair. Nature *513*, 120–123.
16. Findlay, G.M., Daza, R.M., Martin, B., Zhang, M.D., Leith, A.P., Gasperini, M., Janizek, J.D., Huang, X., Starita, L.M., and Shendure, J. (2018). Accurate classification of BRCA1 variants with saturation genome editing. Nature *562*, 217–222.
17. Buckley, M., Kajba, C.M., Forrester, N., Terwagne, C., Sawyer, C., Shepherd, S.T.C., Jonghe, J.D., Dace, P., Turajlic, S., and Findlay, G.M. (2024). Saturation Genome Editing Resolves the Functional Spectrum of Pathogenic VHL Alleles. Nat. Genet. *56*, 1446–1455.
18. Radford, E.J., Tan, H.K., Andersson, M.H.L., Stephenson, J.D., Gardner, E.J., Ironfield, H., Waters, A.J., Gitterman, D., Lindsay, S., Abascal, F., et al. (2023). Saturation genome editing of DDX3X clarifies pathogenicity of germline and somatic variation. Nat. Commun. *14*, 7702.
19. Meitlis, I., Allenspach, E.J., Bauman, B.M., Phan, I.Q., Dabbah, G., Schmitt, E.G., Camp, N.D., Torgerson, T.R., Nickerson, D.A., Bamshad, M.J., et al. (2020). Multiplexed Functional Assessment of Genetic Variants in CARD11. Am. J. Hum. Genet. *107*, 1029–1043.
20. Hanna, R.E., Hegde, M., Fagre, C.R., DeWeirdt, P.C., Sangree, A.K., Szegletes, Z., Griffith, A., Feeley, M.N., Sanson, K.R., Baidi, Y., et al. (2021). Massively parallel assessment of human variants with base editor screens. Cell *184*, 1064–1080.e20.
21. Martin-Rufino, J.D., Castano, N., Pang, M., Grody, E.I., Joubran, S., Caulier, A., Wahlster, L., Li, T., Qiu, X., Riera-Escandell, A.M., et al. (2023). Massively parallel base editing to map variant effects in human hematopoiesis. Cell *186*, 2456–2474.e24.
22. Anzalone, A.V., Randolph, P.B., Davis, J.R., Sousa, A.A., Koblan, L.W., Levy, J.M., Chen, P.J., Wilson, C., Newby, G.A., Raguram, A., and Liu, D.R. (2019). Search-and-replace genome editing without double-strand breaks or donor DNA. Nature *576*, 149–157.
23. Ren, X., Yang, H., Nierenberg, J.L., Sun, Y., Chen, J., Beaman, C., Pham, T., Nobuhara, M., Takagi, M.A., Narayan, V., et al. (2023). High throughput PRIME editing screens identify functional DNA variants in the human genome. Mol. Cell *83*, 4633–4645.e9.
24. Erwood, S., Bily, T.M.I., Lequyer, J., Yan, J., Gulati, N., Brewer, R.A., Zhou, L., Pelletier, L., Ivakine, E.A., and Cohn, R.D. (2022). Saturation variant interpretation using CRISPR prime editing. Nat. Biotechnol. *40*, 885–895.
25. Kim, Y., Oh, H.-C., Lee, S., and Kim, H.H. (2025). Saturation profiling of drug-resistant genetic variants using prime editing. Nat. Biotechnol. *43*, 1471–1484. https://doi.org/10.1038/s41587-024-02465-z.
26. Lee, K.S., Min, J.-G., Cheong, Y., Oh, H.-C., Jung, S.-Y., Park, J.-I., Song, M., Seo, J.H., Cho, S.-R., and Kim, H.H. (2025). Functional assessment of all ATM SNVs using prime editing and deep learning. Cell *188*, 5081–5099.e27. https://doi.org/10.1016/j.cell.2025.05.046.
27. Ren, X., Yang, H., Nierenberg, J.L., Sun, Y., Chen, J., Beaman, C., Pham, T., Nobuhara, M., Takagi, M.A., Narayan, V., et al. (2023). High-throughput PRIME-editing screens identify functional DNA variants in the human genome. Mol. Cell *83*, 4633–4645.e9.
28. Gould, S.I., Wuest, A.N., Dong, K., Johnson, G.A., Hsu, A., Narendra, V.K., Levine, S.S., Liu, D.R., and Sánchez Rivera, F.J. (2025). High throughput evaluation of genetic variants with prime editing sensor libraries. Nat. Biotechnol. *43*, 1648–1662.
29. Herger, M., Kajba, C.M., Buckley, M., Cunha, A., Strom, M., and Findlay, G.M. (2025). High-throughput screening of human genetic variants by pooled prime editing. Cell Genom. *5*, 100814.
30. Yosaatmadja, Y., Silva, S., Dickson, J.M., Patterson, A.V., Smaill, J.B., Flanagan, J.U., McKeage, M.J., and Squire, C.J. (2015). Binding mode of the breakthrough inhibitor AZD9291 to epidermal growth factor receptor revealed. J. Struct. Biol. *192*, 539–544.
31. Thress, K.S., Paweletz, C.P., Felip, E., Cho, B.C., Stetson, D., Dougherty, B., Lai, Z., Markovets, A., Vivancos, A., Kuang, Y., et al. (2015). Acquired EGFR C797S mutation mediates resistance to AZD9291 in non–small cell lung cancer harboring EGFR T790M. Nat. Med. *21*, 560–562.
32. Cross, D.A.E., Ashton, S.E., Ghiorghiu, S., Eberlein, C., Nebhan, C.A., Spitzler, P.J., Orme, J.P., Finlay, M.R.V., Ward, R.A., Mellor, M.J., et al. (2014). AZD9291, an irreversible EGFR TKI, overcomes T790M-mediated resistance to EGFR inhibitors in lung cancer. Cancer Discov. *4*, 1046–1061.
33. Ettinger, D.S., Wood, D.E., Aisner, D.L., Akerley, W., Bauman, J.R., Bharat, A., Bruno, D.S., Chang, J.Y., Chirieac, L.R., DeCamp, M., et al. (2023). NCCN Guidelines® Insights: Non–Small Cell Lung Cancer, Version 2.2023: Featured Updates to the NCCN Guidelines. J. Natl. Compr. Cancer Netw. *21*, 340–350.
34. Park, M.-Y., Jung, M.H., Eo, E.Y., Kim, S., Lee, S.H., Lee, Y.J., Park, J.S., Cho, Y.J., Chung, J.H., Kim, C.H., et al. (2017). Generation of lung cancer cell lines harboring EGFR T790M mutation by CRISPR/Cas9-mediated genome editing. Oncotarget *8*, 36331–36338.
35. Hata, A.N., Niederst, M.J., Archibald, H.L., Gomez-Caraballo, M., Siddiqui, F.M., Mulvey, H.E., Maruvka, Y.E., Ji, F., Bhang, H.-E.C., Krishnamurthy Radhakrishna, V., et al. (2016). Tumor cells can follow distinct evolutionary paths to become resistant to epidermal growth factor receptor inhibition. Nat. Med. *22*, 262–269.
36. Panda, S.K., Wigerblad, G., Jiang, L., Jiménez-Andrade, Y., Iyer, V.S., Shen, Y., Boddul, S.V., Guerreiro-Cacais, A.O., Raposo, B., Kasza, Z., and Wermeling, F. (2020). IL-4 controls activated neutrophil FcγR2b expression and migration into inflamed joints. Proc. Natl. Acad. Sci. USA *117*, 3103–3113.
37. Nelson, J.W., Randolph, P.B., Shen, S.P., Everette, K.A., Chen, P.J., Anzalone, A.V., An, M., Newby, G.A., Chen, J.C., Hsu, A., and Liu, D.R. (2022). Engineered pegRNAs improve prime editing efficiency. Nat. Biotechnol. *40*, 402–410.
38. Chen, P.J., Hussmann, J.A., Yan, J., Knipping, F., Ravisankar, P., Chen, P.-F., Chen, C., Nelson, J.W., Newby, G.A., Sahin, M., et al. (2021). Enhanced prime editing systems by manipulating cellular determinants of editing outcomes. Cell *184*, 5635–5652.e29.

CellPress
OPEN ACCESS

39. Cancer Genome Atlas Research Network (2014). Comprehensive molecular profiling of lung adenocarcinoma. Nature *511*, 543–550.

40. Fowler, D.M., and Fields, S. (2014). Deep mutational scanning: a new style of protein science. Nat. Methods *11*, 801–807.

41. Jänne, P.A., Yang, J.C.-H., Kim, D.-W., Planchard, D., Ohe, Y., Ramalingam, S.S., Ahn, M.-J., Kim, S.-W., Su, W.-C., Horn, L., et al. (2015). AZD9291 in EGFR Inhibitor–Resistant Non–Small-Cell Lung Cancer. N. Engl. J. Med. *372*, 1689–1699.

42. Wang, M., Yang, J.C.-H., Mitchell, P.L., Fang, J., Camidge, D.R., Nian, W., Chiu, C.-H., Zhou, J., Zhao, Y., Su, W.-C., et al. (2022). Sunvozertinib, a Selective EGFR Inhibitor for Previously Treated Non-Small Cell Lung Cancer with EGFR Exon 20 Insertion Mutations. Cancer Discov. *12*, 1676–1689.

43. Kashima, K., Kawauchi, H., Tanimura, H., Tachibana, Y., Chiba, T., Torizawa, T., and Sakamoto, H. (2020). CH7233163 Overcomes Osimertinib-Resistant EGFR-Del19/T790M/C797S Mutation. Mol. Cancer Therapeut. *19*, 2288–2297.

44. Du, X., Yang, B., An, Q., Assaraf, Y.G., Cao, X., and Xia, J. (2021). Acquired resistance to third-generation EGFR-TKIs and emerging next-generation EGFR inhibitors. Innovation *2*, 100103.

45. Love, M.I., Huber, W., and Anders, S. (2014). Moderated estimation of fold change and dispersion for RNA-seq data with DESeq2. Genome Biol. *15*, 550.

46. Huang, L., Guo, Z., Wang, F., and Fu, L. (2021). KRAS mutation: from undruggable to druggable in cancer. Signal Transduct. Targeted Ther. *6*, 386.

47. Skoulidis, F., Li, B.T., Dy, G.K., Price, T.J., Falchook, G.S., Wolf, J., Italiano, A., Schuler, M., Borghaei, H., Barlesi, F., et al. (2021). Sotorasib for Lung Cancers with KRAS p.G12C Mutation. N. Engl. J. Med. *384*, 2371–2381.

48. Hunter, J.C., Manandhar, A., Carrasco, M.A., Gurbani, D., Gondi, S., and Westover, K.D. (2015). Biochemical and Structural Analysis of Common Cancer-Associated KRAS Mutations. Mol. Cancer Res. *13*, 1325–1335.

49. Mathis, N., Allam, A., Tálas, A., Kissling, L., Benvenuto, E., Schmidheini, L., Schep, R., Damodharan, T., Balázs, Z., Janjuha, S., et al. (2025). Machine learning prediction of prime editing efficiency across diverse chromatin contexts. Nat. Biotechnol. *43*, 712–719.

50. Guibert, N., Hu, Y., Feeney, N., Kuang, Y., Plagnol, V., Jones, G., Howarth, K., Beeler, J.F., Paweletz, C.P., and Oxnard, G.R. (2018). Amplicon-based next-generation sequencing of plasma cell-free DNA for detection of driver and resistance mutations in advanced non-small cell lung cancer. Ann. Oncol. *29*, 1049–1055.

51. Li, C., Wang, Y., Su, K., Liu, Y., Wang, L., Zheng, B., Yan, N., Yuan, D., Zhang, Y., Xue, L., et al. (2021). Presentation of EGFR mutations in 162 family probands with multiple primary lung cancer. Transl. Lung Cancer Res. *10*, 1734–1746.

52. Lin, L., Lu, Q., Cao, R., Ou, Q., Ma, Y., Bao, H., Wu, X., Shao, Y., Wang, Z., and Shen, B. (2020). Acquired rare recurrent EGFR mutations as mechanisms of resistance to Osimertinib in lung cancer and in silico structural modelling. Am. J. Cancer Res. *10*, 4005–4015.

53. Boldrini, L., Alì, G., Gisfredi, S., Ursino, S., Baldini, E., Melfi, F., Lucchi, M., Comin, C.E., Maddau, C., Tibaldi, C., et al. (2009). Epidermal growth factor receptor and K-RAS mutations in 411 lung adenocarcinoma: a population-based prospective study. Oncol. Rep. *22*, 683–691.

54. Foster, J.M., Radhakrishna, U., Govindarajan, V., Carreau, J.H., Gatalica, Z., Sharma, P., Nath, S.K., and Loggie, B.W. (2010). Clinical implications of novel activating EGFR mutations in malignant peritoneal mesothelioma. World J. Surg. Oncol. *8*, 88.

55. Liu, Z., Liu, L., Li, M., Wang, Z., Feng, L., Zhang, Q., Cheng, S., and Lu, S. (2011). Epidermal growth factor receptor mutation in gastric cancer. Pathology *43*, 234–238.

56. Na, I.I., Kang, H.J., Cho, S.Y., Koh, J.S., Lee, J.K., Lee, B.C., Lee, G.H., Lee, Y.S., Yoo, H.J., Ryoo, B.-Y., et al. (2007). EGFR mutations and human papillomavirus in squamous cell carcinoma of tongue and tonsil. Eur. J. Cancer *43*, 520–526.

57. Liu, Y., Wu, B.-Q., Zhong, H.-H., Hui, P., and Fang, W.-G. (2013). Screening for EGFR and KRAS mutations in non-small cell lung carcinomas using DNA extraction by hydrothermal pressure coupled with PCR-based direct sequencing. Int. J. Clin. Exp. Pathol. *6*, 1880–1889.

58. Wang, Y., Hao, Y., Ranieri, M., Abramyan, T.M., Tsidilkovski, L., Hollenberg, M., Lopez, A., Moore, X.T.R., Sherman, F., Deng, J., et al. (2025). Deep mutational scanning reveals EGFR mutations conferring resistance to the 4th-generation EGFR tyrosine kinase inhibitor BLU-945. npj Precis. Oncol. *9*, 294.

59. Hsu, J.Y., Grünewald, J., Szalay, R., Shih, J., Anzalone, A.V., Lam, K.C., Shen, M.W., Petri, K., Liu, D.R., Joung, J.K., and Pinello, L. (2021). PrimeDesign software for rapid and simplified design of prime editing guide RNAs. Nat. Commun. *12*, 1034.

60. Koeppel, J., Weller, J., Peets, E.M., Pallaseni, A., Kuzmin, I., Raudvere, U., Peterson, H., Liberante, F.G., and Parts, L. (2023). Prediction of prime editing insertion efficiencies using sequence features and DNA repair determinants. Nat. Biotechnol. *41*, 1446–1456. https://doi.org/10.1038/s41587-023-01678-y.

61. Choi, J., Chen, W., Suiter, C.C., Lee, C., Chardon, F.M., Yang, W., Leith, A., Daza, R.M., Martin, B., and Shendure, J. (2022). Precise genomic deletions using paired prime editing. Nat. Biotechnol. *40*, 218–226.

STAR★METHODS

KEY RESOURCES TABLE

REAGENT or RESOURCE	SOURCE	IDENTIFIER
**Antibodies**		
MLH1 Monoclonal Antibody	Invitrogen	MA5-15431; RRID: AB_10981391
Goat anti-Mouse IgG (H + L) Secondary Antibody, HRP	Invitrogen	31430; RRID: AB_228307
**Bacterial and virus strains**		
Stable competent E. coli cells	New England Biolabs	C3040H
Electrocompetent E. coli cells	New England Biolabs	C3020K
**Chemicals, peptides, and recombinant proteins**		
6-TG	Sigma Aldrich	A4882
Piggybac transposase	System Biosciences	PB210PA-1
DMSO	Sigma Aldrich	D2650
osimertinib	Selleck Chemicals	S7297
CH7233163	Selleck Chemicals	S9711
sunvozertinib	Selleck Chemicals	E0368
Vemurafenib	Selleck Chemicals	S1267
**Deposited data**		
All deposited data for this manuscript can be found at https://krishna.gs.washington.edu/content/members/multiplex_PE_screening/public/ and on Figshare: https://figshare.com/projects/A_multiplex_prime_editing_framework_for_identifying_drug_resistance_variants_at_scale/247232	This paper	N/A
**Experimental models: Cell lines**		
PC-9 cells	Dr. Mathew Meyerson (Broad Institute)	N/A
A-375 cells	ATCC	CRL-1619
MLH1ko-PEMax-PC-9	This paper	N/A
MLH1ko-PEmax-A375	This paper	N/A
HEK293T	ATCC	CRL-3216
**Experimental models: Organisms/strains**		
Nu/J immunocompromised mice	JAX	02019
**Oligonucleotides**		
Oligos for EGFR C797S arrayed editing experiments, see Table S1	This paper	N/A
Locus-specific PCR amplification primers, see Table S2	This paper	N/A
Sequences ordered in Twist oligo pool for small scale screen with 121 epegRNAs, see Table S4	This paper	N/A
Sequences ordered in Twist oligo pool for large scale screens with 3,825 epegRNA, see Table S10	This paper	N/A
pegRNAs designed with PrimeDesign and PRIDICT 2.0, see Table S16	This paper	N/A
All other oligonucleotides are described in the Methods section of the paper, see STAR Methods	This paper	N/A
**Recombinant DNA**		
PB-EFS-PEmax	This paper	N/A
pCMV-PEmax	Addgene	174820
Piggybac-PE2-puro	Choi et al.[61]	N/A
PiggyBac-PE2-Blast	This paper	N/A

(*Continued on next page*)

Continued		
REAGENT or RESOURCE	SOURCE	IDENTIFIER
pSpCas9(BB)-2A-Puro (PX459) V2.0	Addgene	62988
pU6-pegRNA-GG-acceptor	Addgene	132777
LentiGuide-Puro-P2A-EGFP	Addgene	137729
Lenti-epeg-Puro-P2A-EGFP	This paper	N/A
Software and algorithms		
PrimeDesign	https://github.com/pinellolab/PrimeDesign	N/A
PRIDICT	https://github.com/uzh-dqbm-cmi/PRIDICT2	N/A
Bcl2fastq version 2.20	Illumina	N/A
DESeq2	https://github.com/thelovelab/DESeq2	N/A
Custom code used in this paper can be found at https://figshare.com/projects/A_multiplex_prime_editing_framework_for_identifying_drug_resistance_variants_at_scale/247232	This paper	N/A
Other		
NEBuilder HiFi DNA Assembly Master Mix	New England Biolabs	E2621S
Monarch Plasmid Miniprep Kit	New England Biolabs	T1010L
transIT-LT1	Mirius Bio	MIR 2300
BsaI-HFv2	New England Biolabs	R3733S
Gel extraction kit	New England Biolabs	T1020S
T4 DNA ligase	New England Biolabs	B0202S
Plasmid Miniprep Kit	Zymo Research	D4208T
BsmBI-v2	New England Biolabs	R0739S
Qiagen Plasmid Midi Kit	Qiagen	12143
Virapower lentiviral packaging mix	ThermoFisher Scientific	K497500
Lipofectamine 3000	ThermoFisher Scientific	L3000001
Opti-MEM reduced serum media	ThermoFisher Scientific	31985062
PEG-it virus precipitation solution	System Biosciences	LV810A-1
SF Cell Line 4D-Nucleofector X Kit S	Lonza	V4XC-2032
Nunc EasyFill Cell Factories	ThermoFisher Scientific	140360
Puregene Cell Kit	Qiagen	158767
GlycoBlue coprecipitant	ThermoFisher Scientific	AM9515
KAPA2G Robust HotStart ReadyMix	Roche Diagnostics	KK5702
1X AMpure XP beads	Beckman Coulter	A63880
KAPA HiFi HotStart ReadyMix	Roche Diagnostics	KK2602

EXPERIMENTAL MODEL AND STUDY PARTICIPANT DETAILS

Cell lines and culture

PC-9 cell culture

PC-9 cells (gift from Dr. Matthew Meyerson (Broad Institute)) were originally derived from a metastatic lung adenocarcinoma from a 45 year old male patient. All PC-9 cells were grown at 37°C, and cultured in RPMI 1640 + L-Glutamine (GIBCO, Cat. No. 11-875-093) supplemented with 10% fetal bovine serum (Fisher Scientific, Cat No. SH3039603) and 1% penicillin-streptomycin (Thermo Fisher Scientific, Cat. No. 15070063).

A-375 cell culture

A-375 cells (ATCC, Cat. No. CRL-1619) were originally derived from a malignant melanoma from the skin of a 54 year old female patient. All A-375 cells were grown at 37°C and cultured in DMEM, high glucose (Gibco, Cat. No. 11995-040) supplemented with 10% fetal bovine serum (Sigma-Aldrich, Cat. No. F0926) and 1% penicillin-streptomycin (Thermo Fisher Scientific Cat. No. 15070063).

METHOD DETAILS

Piggybac-PEmax vector cloning

The PB-EFS-PEmax vector was constructed as follows. The PEmax coding sequence from the T7 promoter to downstream of the bGH-PolyA tail was amplified out of the pCMV-PEmax plasmid (Addgene #174820) with the following primers CGCCAGAACACAGGACCGGTTAATACGACTCACTATAGGGAGAG (forward primer) and AGCGATCGCAGATCCTTCGCTAATGT GAGTTAGCTCACTCATT (reverse primer). An inverse PCR amplification was done of the backbone of the PiggyBac-PE2-Blast plasmid (generated by exchanging the puromycin resistance cassette in the Piggybac-PE2-puro plasmid[61] with a blasticidin resistance cassette) with the following primers: CCCTATAGTGAGTCGTATTAGGTGGCAGCGCTCTAGAACC (forward primer) and GAGTGAGCTAACTCACATTACTTCTGAGGCGGAAAGAACC (reverse primer). These two amplification products were then assembled into a single vector (PB-EFS-PEmax) using NEBuilder HiFi DNA Assembly Master Mix (New England Biolabs, Cat. No. E2621S) using the standard protocol for a 2–3 fragment assembly. 1 μL of the 20 μL assembly reaction was transformed into 50 μL of stable competent E. coli cells (New England Biolabs, Cat. No. C3040H) using the NEB 5 min transformation protocol. 100 μL of transformed E. coli cells was plated on an LB agar plate containing ampicillin, and single colonies were picked 1 day later to grow up and extract plasmid DNA using a Monarch Plasmid Miniprep Kit (New England Biolabs, Cat. No. T1010L). Extracted plasmid DNA was sequence confirmed via long-read Nanopore sequencing (Primordium Labs) and DNA from a single clone harboring the correct assembled sequence was used for all experiments.

MLH1 knockout-PEmax cell line generation and validation in PC-9 cells

MLH1 *knockout, selection, and single-cell sorting*

MLH1 was knocked out of a population of PC-9 cells using a single gRNA targeting exon 2 of *MLH1*. The sequence of this gRNA is AAGACAATGGCACCGGGATC. This gRNA was cloned into pSpCas9(BB)-2A-Puro (PX459) V2.0 (Addgene #62988) via the Zhang lab protocol (https://media.addgene.org/data/plasmids/62/62988/62988-attachment_KsK1asO9w4owD8K6wp8.pdf). 2.5 μg of assembled vector was transiently transfected into 250,000 wild type PC-9 cells using the transIT-LT1 transfection reagent (Mirius Bio, Cat. No. MIR 2300). 2 days after transfection, 1 μg/mL concentration of puromycin (GIBCO/Thermo Fisher Scientific, Cat. No. A1113803) was added to cells to select for successfully transfected cells over a period of 4 days.

After puromycin selection, this population of cells was single-cell sorted into 96-well plates to grow up clonal cell lines. 12 clonal lines were expanded, split into two sets of parallel cultures (i.e., 24 wells with 2 wells per clonal line), and one set was treated with 1.5 μM 6-TG (Sigma Aldrich, Cat. No. A4882) for 4 days to screen for cells with *MLH1* successfully knocked out. 5 of 12 treated wells survived 6-TG treatment (denoted clones A, B, C, D, and E). PCR primers targeting *MLH1* (forward primer: TGTATGAG CCTGTAAGACAAAGGAA, reverse primer: CATCCATATTGAAGCCTTCCTGAAC were used on extracted gDNA from these 5 clonal lines to amplify the *MLH1* locus and confirm knockout via sanger sequencing. A western blot was performed on 3 of the monoclonal lines to confirm knockout (primary antibody used: MLH1 Monoclonal Antibody; Invitrogen, Cat No. MA5-15431, secondary antibody used: Goat anti-Mouse IgG (H + L) Secondary Antibody, HRP; Invitrogen, Cat. No. 31430), and complete loss of the MLH1 protein was confirmed for two clones (A and E) (Figure S4). Clones A and E were chosen for further cell line engineering. Clone E, was ultimately used for all experiments.

Prime editor-max (PEmax) transfection, selection, and single-cell sorting

Clones A and E were transfected with a Piggybac-PEmax plasmid with the Piggybac transposase (System Biosciences, Cat. No. PB210PA-1) at a 10:1 molar ratio using the transIT-LT1 transfection reagent (Mirius Bio, Cat. No. MIR 2300). 2.5 μg of total DNA (Piggybac-PEmax plasmid, and the transposase plasmid), along with 5 μL of *trans*-IT reagent was reverse-transfected into 100,000 cells for each well of a 12-well plate. Cells were selected with 10 μg/mL of blasticidin for 10 days to select for cells that successfully integrated the PEmax construct. These polyclonal cells were single-cell sorted into 96-well plates to grow up clonal cell lines to generate a MLH1ko-PEMax-PC-9 cell line.

Prime editing experiment to assess editing efficiencies of 15 monoclonal lines

15 monoclonal lines were expanded, and prime editing efficiency of 12 of these lines were tested by performing an arrayed experiment in which we tested for the ability of these cells to insert a trinucleotide sequence (CTT) at the *HEK3* locus when transfected with a pegRNA that programs this insertion[22] (Figure S4). From this experiment, we chose to use Clone A6 for all further prime editing experiments. While Clones A8 and A12 exhibited higher editing efficiency (Figure S4), these clones grew poorly and were not considered for future experiments.

MLH1 knockout-PEmax cell line generation and validation in A-375 cells

We engineered MLH1ko-PEmax-A375 in the same manner as MLH1ko-PEmax-PC-9. We transfected 2.5 μg of the same *MLH1* exon2 targeting gRNA into 250,000 wild type A375 cells with Lipofectamine 3000 (Thermo Fisher Scientific, Cat. No. L3000001) according to the manufacturer's protocol. Puromycin selection, single-cell sorting, 6-TG screening, PCR, and Sanger sequencing were performed in the same manner as described for PC-9 cells. A western blot was performed on 4 candidate clones, 3 of which demonstrated complete loss of the MLH1 protein (primary antibody used: MLH1 Monoclonal Antibody; Invitrogen, Cat No. MA5-15431, secondary antibody used: Goat anti-Mouse IgG (H + L) Cross-Adsorbed Secondary Antibody, Alexa Fluor 488, Thermo Fisher Scientific, Cat. No. A-11001). Clone 9 was transfected with the same Piggybac-PEmax and

transposase plasmids with Lipofectamine 3000 according to the manufacturer's protocol. Blasticidin selection and single-cell sorting was performed in the same manner as with the PC-9 cells. 6 clonal lines were expanded and the prime editing efficiency of the same CTT insertion at the *HEK3* locus was assessed. Clone E was selected for all experiments and established as MLH1ko-PEmax-A375.

pegRNA selection and design

All pegRNAs were designed using either the PrimeDesign[59] or PRIDICT 2.0 web or command line interface, using default parameters. Up to four pegRNAs were designed for each individual programmed edit. Input files used for designing pegRNAs and designed pegRNAs are in Tables S3, S9, and S16.

pegRNA cloning into transient and lentiviral vectors

EGFR C797S T>A transiently transfected editing experiments

For the EGFR transient C797S T>A editing experiments, the following three pegRNA containing oligos (denoted pegRNA_1, pegRNA_2, and pegRNA_3) were ordered as three separate oPools from Integrated DNA Technologies (IDT).

1) pegRNA_1: CACCG**ATCACGCAGCTCATGCCCTT**GTTTTAGAGCTAGAAATAGCAAGTTAAAATAAGGCTAGTCCGTTATCAACTTGAAAAAGTGGGACCGAGTCGGTCC**TCCAGGAGGC*T*GCCGAAGGGCATGAGCTGC**TTTT
2) pegRNA_2: CACC**GTTCCCGGACATAGTCCAGG**GTTTTAGAGCTAGAAATAGCAAGTTAAAATAAGGCTAGTCCGTTATCAACTTGAAAAAGTGGGACCGAGTCGGTCC**TTCGGC*A*GCCTCCTGGACTATGTCCGG**TTTT
3) pegRNA_3: CACCG**TGTGTTCCCGGACATAGTCC**GTTTTAGAGCTAGAAATAGCAAGTTAAAATAAGGCTAGTCCGTTATCAACTTGAAAAAGTGGGACCGAGTCGGTCC**TTCGGC*A*GCCTCCTGGACTATGTCCGGGAA**TTTT

20 base pair spacer sequences and variable length extension sequences are in bold. Programmed single nucleotide edit are in bold and italics.

The pU6-pegRNA-GG-acceptor (Addgene plasmid #132777) was digested with BsaI-HFv2 (New England Biolabs, Cat. No. R3733S) in 10X rCutSmart Buffer at 37°C overnight to ensure complete digestion of the backbone. This digestion cuts out the mRFP1 cassette (821 base pairs). The linear backbone vector (2,183 base pairs in size) was gel extracted using a gel extraction kit (New England Biolabs, Cat. No. T1020S) and assembled with the pegRNA oligos (listed above) via Golden Gate assembly using the following amounts: 30 ng of linearized backbone, 1 μL of 1 μM pegRNA oligo, 0.25 μL of BsaI-HFv2 (New England Biolabs, Cat. No. R3733S), 0.5 μL of T4 DNA ligase (New England Biolabs, Cat. No. M0202S) and 1 μL of 10X T4 DNA ligase reaction buffer (New England Biolabs, Cat. No. B0202S) in a final volume of 10 μL. 1 μL of the assembly reaction was transformed into 50 μL of stable competent E. coli cells (New England Biolabs, Cat. No. C3040H) and plated on an LB agar plate containing ampicillin, and single colonies miniprepped and used for transfection (Zymo Research, Cat. No. D4208T).

EGFR C797S T>A arrayed and 121 epegRNA pooled lentiviral editing screens

For the EGFR lentiviral C797S T>A editing experiments, the following three pegRNA-containing oligos (denoted lenti_epegRNA_1, lenti_epegRNA_2, and lenti_epegRNA _3) were ordered as three separate oPools from Integrated DNA Technologies (IDT).

1) lenti_epegRNA_1: gctttatatatcttgtggaaaggacgaaacacc**GATCACGCAGCTCATGCCCTT**gttttagagctagaaatagcaagttaaaataaggctagtccgttatcaacttgaaaaagtggGaccgagtcggtCc**TCCAGGAGGC*T*GCCGAAGGGCATGAGCTGC**TTGACGCGGTTCTATCTAGTTACGCGTTAAACCAACTAGAAAtttttttNNNNNNNNggagacgaagcttggcg
2) lenti_epegRNA_2: gctttatatatcttgtggaaaggacgaaacacc**GGTTCCCGGACATAGTCCAGG**gttttagagctagaaatagcaagttaaaataaggctagtccgttatcaacttgaaaaagtggGaccgagtcggtCc**TTCGGC*A*GCCTCCTGGACTATGTCCGG**TTGACGCGGTTCTATCTAGTTACGCGTTAAACCAACTAGAAAtttttttNNNNNNNNggagacgaagcttggcg
3) lenti_epegRNA_3: gctttatatatcttgtggaaaggacgaaacacc**GTGTGTTCCCGGACATAGTCC**gttttagagctagaaatagcaagttaaaataaggctagtccgttatcaacttgaaaaagtggGaccgagtcggtCc**TTCGGC*A*GCCTCCTGGACTATGTCCGGGAA**TTGACGCGGTTCTATCTAGTTACGCGTTAAACCAACTAGAAAtttttttNNNNNNNNggagacgaagcttggcg

20 base pair spacer sequences and variable length extension sequences are in bold. Programmed single nucleotide edit are in bold and italics.

The LentiGuide-Puro-P2A-EGFP (Addgene plasmid #137729) was modified to enable cloning of epegRNAs via Gibson assembly. The modified plasmid, which we term Lenti-epeg-Puro-P2A-EGFP, was used for cloning all epegRNAs. Lenti-epeg-Puro-P2A-EGFP was digested with BsmBI-v2 (New England Biolabs, Cat. No. R0739S) to create a single cut and produce a linearized backbone vector of 9007 base pairs. The three lenti_epegRNA oligos were PCR amplified with the following primers: gctttatatatcttgtggaaaggacg (forward primer) and cgccaagcttcgtctcc (reverse primer) to create double stranded DNA. The double-stranded lenti_epegRNA oligos were then assembled with the linearized Lenti-epeg-Puro-P2A-EGFP backbone using NEBuilder HiFi DNA Assembly Master Mix (New England Biolabs, Cat. No. E2621S) using the standard protocol. 1 μL of the 20 μL assembly reaction was transformed into 50 μL of stable competent E. coli cells (New England Biolabs, Cat. No. C3040H), plated on an LB agar plate containing ampicillin, and single colonies were miniprepped (Zymo Research, Cat. No. D4208T) and used for lentivirus generation.

We observed that lentiviral transduction of epegRNAs resulted in lower editing efficiencies compared to transient transfection of pegRNAs, despite the use of an improved epegRNA scaffold. This difference likely reflects a combination of factors, including shorter selection periods in the lentiviral experiments, lower pegRNA expression due to low multiplicity of infection (MOI), and potential variability from genomic integration site effects. Together, these factors may have outweighed the expected benefits of the engineered pegRNA scaffold under these conditions.

We sequenced the cloned plasmid library, and found that the pegRNAs are all well represented, with some variation as expected. 86 of 121 pegRNAs (71.1%) are within 1 standard deviation of the mean, 18 of 121 (14.9%) are below 1 standard deviation of the mean, and 17 of 121 (14%) are above 1 standard deviation of the mean. All pegRNA spacers were associated with the expected extension sequence, confirming that there was no detachment of RTTs during cloning.

1,220 variant lentiviral pooled editing screen

epegRNA-containing oligos were ordered as four separate sub-libraries in one single oligo pool from Twist biosciences. Sequences in this oligo pool are in Table S2. The four sub-libraries were cloned separately into the Lenti-epeg-Puro-P2A-EGFP vector, following the steps described above for the 121 epegRNA pool, with the only difference being the addition of an 8N degenerate barcode during initial amplification of the Twist oligo pool, and that after assembly, 2 μL of each library was transformed into 50 μL of electrocompetent E. coli cells (New England Biolabs, Cat. No. C3020K) via electroporation. 990 μL of transformed cells were cultured in 50 mL of LB media +100 μg/mL ampicillin at 31C. 10 μL (out of a total volume of 1000 μL) of the transformed E. coli cells was plated on LB agar plates containing ampicillin, and colonies were counted the next day to estimate the number of clones in each library. Transformations were performed for each library until each library had a minimum of 1000X coverage of each epegRNA. After overnight culture at 31C, the epegRNA lentiviral plasmid libraries were extracted using a Qiagen Plasmid Midi Kit (Qiagen, Cat. No. 12143). Extracted plasmid DNA from the four libraries was pooled to generate a pool with 3,825 epegRNAs and used for lentivirus generation.

Lentivirus generation

To generate lentivirus, HEK293T cells (ATCC, Cat. No. CRL-3216) were either plated the day before transfection at 0.7×10^6 cells in a T-25 cell culture flask, or the day of transfection at 1.4×10^6 cells in a T-25 cell culture flask. Virapower lentiviral packaging mix (ThermoFisher Scientific, Cat. No. K497500) was used with Lipofectamine 3000 (ThermoFisher Scientific, Cat. No. L3000001) for transfection into HEK293T cells. 1,500 μL of Opti-MEM reduced serum media (ThermoFisher Scientific, Cat. No. 31985062) was mixed with 42 μL of Lipofectamine 3000 in one tube. 13.5 μg of Virapower lentiviral packaging mix, 4.5 μg of the epegRNA lentiviral plasmid or plasmid library, and 36 μL of P3000 reagent was added to a second tube. The two tubes were combined into a single tube and incubated for 10–20 min at room temperature. 50% of the media was removed from the T-25 flask containing the HEK293T cells (unless the cells were plated the day of, then they were only plated in half the amount of media), and the lipid complex from the single tube was added to the cells. The cells were incubated overnight, and media harvests were taken at 24, 48, and 72 h post-transfection. The lentivirus-containing media was mixed at a 1:4 ratio of PEG-it virus precipitation solution (System Biosciences, Cat. No. LV810A-1) to media, and refrigerated at 4C to concentrate the lentiviral particles. After 96 h, all three harvests (24, 48, and 72 h) were spun down at 1,500xg for 30 min at 4C. The lentivirus was a visible white pellet after this spin. The media above the pellet was aspirated, and the lentivirus was resuspended in 400 μL ice-cold 1X PBS (Invitrogen, Cat. No. 14190-144), aliquoted into 100 μL aliquots, and frozen at −80 for later use. Each lentiviral aliquot was only thawed a single time prior to use to avoid multiple freeze-thaw cycles. For the larger scale screens, all amounts were increased in scale to make more lentivirus in larger cell culture dishes (T-75 cell culture flasks).

Lentivirus titration experiment in 3,825 epegRNA lentiviral pooled editing screens

A titration experiment was performed to determine lentivirus amounts to achieve the desired multiplicity of infection (MOI) for both screens. 100,000 PC-9 cells were seeded into each well of a 12-well plate in 1 mL of RPMI 1640 + L-Glutamine media (ThermoFisher Scientific, Cat. No. 11-875-093). 0, 0.5, 1, 2, 4, and 8 μL of virus was added to each of 2 wells in the 12-well plate (2 replicates per lentivirus amount). 48 h after transduction, varying amounts of GFP were observed in the different conditions (GFP is expressed off the epegRNA vector), indicating successful transduction. MOI was determined by flow cytometry (Figure S4). 3 μL of virus for every 100,000 cells was the condition used for the large scale screen to achieve ~30% GFP positivity, which represents an MOI of ~0.35.

Osimertinib dose titration curves

*MLH1*ko-PEmax PC-9 cells expressing either no epegRNA (termed control cells) or an epegRNA coding (lenti_epegRNA_2) for an EGFR C797S mutation (termed $EGFR^{C797S}$ cells) were seeded in duplicate in 12-well dishes at a density of 50,000 cells per well (approximately 14,300 cells/cm^2) in a total volume of 2 mL. Cells were treated with either vehicle (DMSO, Sigma Aldrich Cat. No. D2650) at a concentration of 500 nM or osimertinib (AZD9291, Selleckchem Cat. No. S7297) at a concentration of 100, 300, or 500 nM. From this point, cells were passaged every 3 days and replated at a density of 50,000 cells per well in the appropriate concentration of either vehicle or osimertinib. To measure growth rate and cumulative population doublings of control versus $EGFR^{C797S}$ cells in vehicle and osimertinib, cells were counted at each passage using a Vi-CELL XR analyzer (Beckman Coulter). The 300 nM osimertinib dose, which led to an appreciable growth rate difference between control and $EGFR^{C797S}$ cells was used for all further experiments (Figure S2).

pegRNA transient transfection or lentiviral transduction into PC-9 cells

Transient transfections

For the EGFR C797S T>A proof of concept experiment, the SF Cell Line 4D-Nucleofector X Kit S (Lonza, Cat. No. V4XC-2032) was used to transfect the three pegRNAs and one no DNA control. 2.5 μg of DNA (the pegRNA plasmid and the pCMV-PE2 plasmid (Addgene #132775) at a 1:2 ratio by mass of pegRNA plasmid:PE2), was transfected into 100,000 PC-9 cells that went into four wells of a 12-well plate. 2 days later, 400 nM osimertinib was added to the cells for all conditions (no drug, and the three separate pegRNAs). 24 days later, cells were harvested and the EGFR C797 locus was amplified and sequenced.

Lentiviral transductions

A ratio of 3 μL of lentivirus was added for every 100,000 cells transduced. Cell culture amounts were scaled as necessary to transduce cells at 2,000X coverage of epegRNAs. One day after transduction, media was aspirated and replenished with fresh media. Two days after lentiviral transduction, 2 μg/mL puromycin (GIBCO/Thermo Fisher Scientific, Cat. No. A1113803) was added to the cells for 4 days to select for successfully transduced cells. The cells were replenished with fresh media with 2 μg/mL puromycin each day during these 4 days. For the arrayed *EGFR* C797S T>A experiment, after puromycin selection, we transiently transfected cells with PB-EFS-PEmax twice on two consecutive days. On the second transfection day, cells were treated with 200 nM osimertinib. Eight days later, cells were harvested and the *EGFR* locus was amplified and sequenced. For the 3,825 epegRNA lentiviral pooled editing screens, two parallel plates were cultured without puromycin selection to enable a FACS analysis one week later to determine the MOI of this screen (Figure S5). After 4 days of puromycin selection, 300 nM of drug (for all other lentiviral editing experiments) was added to each treatment condition (CH7233163, osimertinib, or sunvozertinib), and the same volume of DMSO was added to the no treatment control condition. The day that the drug treatments were added marks Day 0 of the screens.

pegRNA lentiviral transduction into MLH1ko-PEmax-A375 cells

For each replicate, 120 million MLH1ko-PEmax-A375 cells were transduced with the epegRNA lentiviral library at low multiplicity of infection (MOI ~0.1) and selected with 2 μg/mL puromycin (Gibco Cat. No. A1113803) for 10 days to ensure stable integration of the epegRNAs. Approximately 160–180 million cells per replicate were seeded into Nunc EasyFill Cell Factories (ThermoFisher Scientific Cat. No. 140360) and treated with 10 μM vemurafenib (Selleck Chemicals, Cat. No. S1267). Media and vemurafenib were replenished every 3–4 days. Cells were harvested at 10, 17, and 24 days following vemurafenib treatment.

Drug treatments used in screens

Cells were treated with DMSO (SigmaAldrich, Cat. No. D2438) or 100, 300, or 500 nM of CH7233163 (Selleck Chemicals, Cat. No. S9711), osimertinib (Selleck Chemicals, Cat. No. S7297), sunvozertinib (Selleck Chemicals, Cat. No. E0368), or 10 μM vemurafenib (Selleck Chemicals, Cat. No. S1267), depending on the screen. Drugs were initially diluted to 1 μM in DMSO, and all further dilutions were done in PBS (Invitrogen, Cat. No. 14190-144) to reach the desired concentrations.

Mouse xenograft cell injections and tumor growth data collection

All animal experiments were carried out with approval by and in accordance with the ethical guidelines of the Fred Hutchinson Cancer Center Institutional Animal Care and Use Committee (Protocol #50967, PI: Berger). Experiments were performed in the Fred Hutch Comparative Medicine facility, which is fully accredited by the Association for Assessment and Accreditation of Laboratory Animal Care (AAALAC) and complies with all United States Department of Agriculture (USDA), Public Health Service (PHS), Washington State and local area animal welfare regulations. For analysis of drug sensitivity *in vivo*, pools of parental or prime edited PC-9 cells (C797S or a pool of variants, $n = 2$ mice/group x 5 injection sites) were trypsinized and washed in PBS before resuspension in cold PBS on ice. 2×10^6 cells in 200 μL PBS were implanted subcutaneously into the flanks (2/animal) of Nu/J immunocompromised mice (JAX #02019). One day later, treatment was initiated with either 5 mg/kg osimertinib (Selleck Chemicals, Cat. No. S7297) or vehicle control (5% DMSO, 40% PEG300, 5% Tween 80 in water) via oral gavage of 100 μL on a 5 days on/2 days off treatment schedule. The 8 variants in the pool were KRAS G12C, EGFR Y801F, EGFR Y801*, EGFR Q791L, EGFR Q791*, EGFR L777M, EGFR Q791P, EGFR T790M. Tumor growth was measured 3× weekly using a digital caliper.

Cell harvests and genomic DNA extraction for the 3,825 epegRNA lentiviral pooled editing screens

Cells were harvested on day 8 for the lentiviral EGFR C797S T>A proof of concept experiment. Cells were harvested on days 3, 7, 11, 15, and 19 for both of the large scale screens. Cells were harvested such that a minimum of 500X coverage of epegRNAs was retained in the remaining culture. At each harvest, fresh media and drug (DMSO, CH7233163, osimertinib, or sunvozertinib) was added to the passaged cells. The harvested cells were spun down at 400 × g for 5 min, aspirated, and cell pellets were stored at −20C. The Puregene Cell Kit (8×10^8) (Qiagen, Cat. No. 158767) was used for all genomic DNA (gDNA) extractions for the large scale screens using the standard protocol, with a single modification to add 2 μL of GlycoBlue coprecipitant (ThermoFisher Scientific, Cat. No. AM9515) to the DNA pellet.

pegRNA amplicon amplification and sequencing

Amplification of the EGFR C797 locus

For the EGFR C797S T>A proof of concept transient and lentiviral editing experiments, the EGFR locus was PCR amplified with the following primers: GCGTCAGATGTGTATAAGAGACAG**CATCTGCCTCACCTCCACCGTG** (forward primer) and GTGACTGGAGTTCAGACGTGTGCTCTTCCGATCT**ACCAGTTGAGCAGGTACTGGGAGC** (reverse primer). The sequences in bold are the locus-binding part of the primer, and the sequences not in bold contain Nextera and Truseq adapter sequences. KAPA2G Robust HotStart ReadyMix (Roche Diagnostics, Cat. No. KK5702) was used for amplification using the recommended protocol with a 60C annealing temperature and a 30 s extension time. The product of this PCR was cleaned with a 1X AMpure XP bead cleanup (Beckman Coulter, Cat. No. A63880), and eluted in 20 μL of water. 1 μL of cleaned PCR product was used for a second PCR which adds the Illumina P5 and P7 adapter sequences and sample-specific indices. This second PCR was done using the following primers: AATGATACGGCGACCACCGAGATCTACACNNNNNNNNNNTCGTCGGCAGCGTCAGATGTGTATAAGAGACAG (forward primer) and CAAGCAGAAGACGGCATACGAGATNNNNNNNNNNGTGACTGGAGTTCAGACGTGTGCTCTTCCGATCT (reverse primer). 10N sequences denote unique indices for each sample. KAPA2G Robust HotStart ReadyMix (Roche Diagnostics, Cat. No. KK5702) was used for amplification using the recommended protocol with a 60C annealing temperature and a 30 s extension time for this second PCR. The product of this PCR was cleaned with a 1X AMpure XP bead cleanup (Beckman Coulter, Cat. No. A63880), and eluted in 20 μL of water. Amplicon concentration and size was determined using the Qubit (ThermoFisher Scientific) and a Tapestation (Agilent) instrument.

Amplification of the integrated genomic DNA pegRNA construct

For the small (121 epegRNAs) and large (3,825 epegRNAs) scale screens, the genomically integrated epegRNAs were amplified via PCR. This is a two-step PCR, with the first PCR amplifying the epegRNA construct and adding partial Illumina read adapter sequences, and with the second PCR adding the Illumina P5 and P7 adapter sequences and sample-specific indices. The first PCR uses the following primers: GCGTCAGATGTGTATAAGAGACAG**cttgtggaaaGGACGAAACACC** (forward primer) and ACGTGTGCTCTTCCGATCT**tctcaagatctagttacgccaagc** (reverse primer). The sequences in bold are the locus-binding part of the primer, and the sequences not in bold contain Nextera and Truseq adapter sequences. KAPA2G Robust HotStart ReadyMix (Roche Diagnostics, Cat. No. KK5702) was used for the 121 epegRNA small scale screen and KAPA HiFi HotStart ReadyMix (Roche Diagnostics, Cat. No. KK2602) was used for amplification in the large scale screens using the recommended protocol with a 65C annealing temperature for the KAPA2G Robust PCR and the KAPA HiFi PCR, and a 30 s extension time for both protocols. The product of this PCR was cleaned with a 1X AMpure XP bead cleanup (Beckman Coulter, Cat. No. A63880), and eluted in 20 μL of water. 5 μL of cleaned PCR product was used for a second PCR which adds the Illumina P5 and P7 adapter sequences and sample-specific indices. This second PCR was done using the following primers: AATGATACGGCGACCACCGAGATCTACACNNNNNNNNNNTCGTCGGCAGCGTCAGATGTGTATAAGAGACAG (forward primer) and CAAGCAGAAGACGGCATACGAGATNNNNNNNNNNGTGACTGGAGTTCAGACGTGTGCTCTTCCGATCT (reverse primer). 10N sequences denote unique indices for each sample. KAPA2G Robust HotStart ReadyMix (Roche Diagnostics, Cat. No. KK5702) was used for the 121 epegRNA small scale screen and KAPA HiFi HotStart ReadyMix (Roche Diagnostics, Cat. No. KK2602) was used for amplification in the large scale screen using the recommended protocol with a 65C annealing temperature for the KAPA2G Robust PCR and the KAPA HiFi PCR, and a 30 s extension time was used for this second PCR for both protocols. The product of this PCR was cleaned with a 1X AMpure XP bead cleanup (Beckman Coulter, Cat. No. A63880), and eluted in 20 μL of water. Amplicon concentration and size was determined using the Qubit (ThermoFisher Scientific) and a Tapestation (Agilent) instrument.

Amplification of endogenous loci targeted with prime editing gRNAs

For the 121 epegRNA lentiviral pooled experiment, the endogenous locus of each target was PCR amplified and sequenced. Each locus was amplified with locus-specific primers which are listed in Table S5. KAPA2G Robust HotStart ReadyMix (Roche Diagnostics, Cat. No. KK5702) was used for amplification using the recommended protocol with a 60C annealing temperature and a 30 s extension time. The product of this PCR was cleaned with a 1X AMpure XP bead cleanup (Beckman Coulter, Cat. No. A63880), and eluted in 20 μL of water. 1 μL of cleaned PCR product was used for a second PCR which adds the Illumina P5 and P7 adapter sequences and sample-specific indices. This second PCR was done using the following primers: AATGATACGGCGACCACCGAGATCTACACNNNNNNNNNNTCGTCGGCAGCGTCAGATGTGTATAAGAGACAG (forward primer) and CAAGCAGAAGACGGCATACGAGATNNNNNNNNNNGTGACTGGAGTTCAGACGTGTGCTCTTCCGATCT (reverse primer). 10N sequences denote unique indices for each sample. KAPA2G Robust HotStart ReadyMix (Roche Diagnostics, Cat. No. KK5702) was used for amplification using the recommended protocol with a 60C annealing temperature and a 30 s extension time for this second PCR. The product of this PCR was cleaned with a 1X AMpure XP bead cleanup (Beckman Coulter, Cat. No. A63880), and eluted in 20 μL of water. Amplicon concentration and size was determined using the Qubit (ThermoFisher Scientific) and a Tapestation (Agilent) instrument.

Sequencing of prime editing screening libraries

Final libraries were sequenced on either a Miseq 300 cycle kit or a NextSeq 2000 P3 200 cycle kit with standard Illumina Nextera and Truseq adapter sequences. The pegRNA spacer was sequenced on Read 1, and the prime editing gRNA extension sequence was sequenced on Read 2. 10 bp index sequences were sequenced with 10 cycle index 1 and index 2 reads.

QUANTIFICATION AND STATISTICAL ANALYSIS

Raw data processing and quality control filtering of sequencing data

Fastq file generation, pegRNA read counting, and Z score calculation

Bcl2fastq version 2.20 was used to generate fastq files using default parameters. Fastq files were then input into count_reads.py via a Snakemake pipeline to count the occurrence of each pegRNA in each day, drug treatment, and replicate condition. A pseudocount of 1 was added to all pegRNA read counts, and then read counts were log2 normalized by condition to normalize for variable sequencing depths across samples. This log2 normalized count matrix was used to calculate the log2 fold change of each pegRNA between each treatment condition and the DMSO control treatment condition. Finally, a *Z* score was calculated for each pegRNA, which is equal to:

$$z - score = \frac{log\,2\left(fold\ change_{pegRNA}\right) - mean\left(log\,2\left(fold\ change\right)_{controls}\right)}{standard\ deviation\left(log\,2\left(fold\ change\right)_{controls}\right)}$$

Controls are pegRNAs that encode for synonymous edits.

DESeq2 for differential pegRNA abundance analysis

DESeq2 was used to identify differential pegRNA abundances in the A-375 KRAS exon 1 vemurafenib screen and the large PC-9 screen that programmed 1,220 variants with 3,825 epegRNAs in ten oncogenes and treated with three kinase inhibitors. The count matrix that was used as an input to DESeq2 was generated from the raw read counts (i.e., not log2 normalized) from the sequencing data.

For the PC-9 drug screens (osimertinib, sunvozertinib, and CH7233163), statistical testing was performed using the likelihood ratio test (LRT). The full model included the variables time, drug treatment, and drug:time (the interaction terms test for the effect of drug as a function of time), and the reduced model contained only the variable time. The likelihood ratio tests whether the parameters in the full model explain the data significantly better than the reduced model.

For the A-375 vemurafenib KRAS screen, variants were identified based on enrichment over time using a DESeq2 Wald test comparing T3 vs. T0 (one-sided). Variants were considered significant at Benjamini-Hochberg false discovery rate (FDR) < 0.05.

Significance was calculated using biological replicate samples (independent lentiviral transductions; n=3), not read coverage alone, thus accounting for variation across replicates. DESeq2 was run using default parameters, except minReplicatesForReplace = Inf in DESeq() and cooksCutoff = FALSE and independentFiltering = FALSE in results(), to retain high-count variants associated with strong resistance phenotypes (e.g., EGFR C797S and KRAS G12 variants). These parameter adjustments were appropriate for variant enrichment screening rather than gene expression analysis (which DESeq2 is typically used for).

All further downstream analyses were done using custom Python and R scripts, which are all accessible on the following website: https://figshare.com/projects/A_multiplex_prime_editing_framework_for_identifying_drug_resistance_variants_at_scale/247232.

Cell Genomics, Volume *6*

Supplemental information

A multiplex, prime editing framework

for identifying drug resistance variants at scale

Florence M.C. Abadie, Chase C. Suiter, Nahum T. Smith, Riza M. Daza, Mary C. Rominger, Phoebe Parrish, Troy A. McDiarmid, Jean-Benoît Lalanne, Beth Martin, Diego Calderon, Amira Ellison, Alice H. Berger, Jay Shendure, and Lea M. Starita

Supplemental Information

A multiplex, prime editing framework for identifying drug resistance variants at scale

Florence M. C. Abadie[1,2,†,*], Chase C. Suiter[1,2,3,†], Nahum T. Smith[4], Riza M. Daza[1], Mary C. Rominger[5], Phoebe Parrish[1,5], Troy A. McDiarmid[1], Jean-Benoît Lalanne[1], Beth Martin[1], Diego Calderon[1,6], Amira Ellison[7], Alice H. Berger[1,5,*], Jay Shendure[1,2,4,8,9,*], Lea M. Starita[1,4,10*]

Affiliations:
1. Department of Genome Sciences, University of Washington, Seattle, WA, 98195, USA
2. Seattle Hub for Synthetic Biology, Seattle, WA, 98109, USA
3. Molecular and Cellular Biology Program, University of Washington, Seattle, WA, 98195, USA
4. Brotman Baty Institute for Precision Medicine, Seattle, WA, 98195, USA
5. Fred Hutchinson Cancer Research Center, Seattle, WA, 98109, USA
6. Bioengineering and Therapeutic Sciences, University of California San Francisco, CA, 94143, USA
7. Department of Molecular and Human Genetics, Baylor College of Medicine, Houston, TX, 77030, USA
8. Howard Hughes Medical Institute, Seattle, WA, 98195, USA
9. Allen Discovery Center for Cell Lineage Tracing, Seattle, WA, 98109, USA
10. Lead contact

† These authors contributed equally to this work

* Correspondence to F.M.C.A. (florence.chardon@alleninstitute.org), A.H.B. (ahberger@fredhutch.org), J.S. (shendure@uw.edu), or L.S. (lstarita@uw.edu)

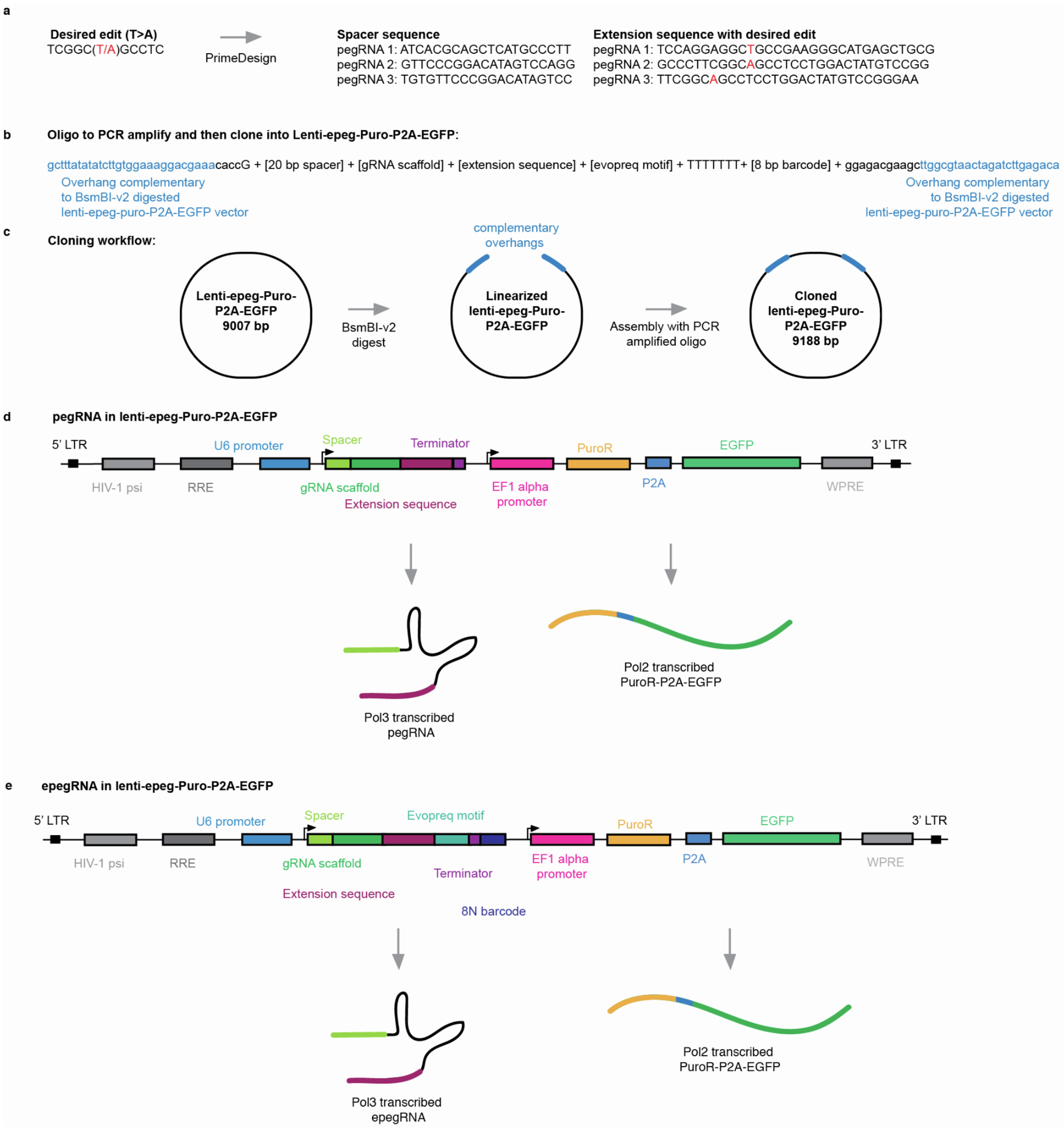

Figure S1 | pegRNA design, cloning, and expression vector components. Related to STAR methods.

a) Schematic of workflow to design pegRNAs with PrimeDesign[57]. Desired edit is in red. **b)** DNA sequence (oligo) to amplify and clone into lenti-epeg-Puro-P2A-EGFP. **c)** Schematic of cloning workflow. Lenti-epeg-Puro-P2A-EGFP is digested with BsmBI-v2, and the PCR amplified oligo from **b)** is assembled with the linearized vector via a Gibson assembly reaction. **d)** Schematic of the lenti-epeg-Puro-P2A-EGFP vector with a pegRNA cloned into it. **e)** Schematic of the lenti-epeg-Puro-P2A-EGFP vector with an epegRNA cloned into it (the epegRNA contains an evopreq RNA stabilizing motif and an 8N barcode sequence 3' of the terminator sequence).

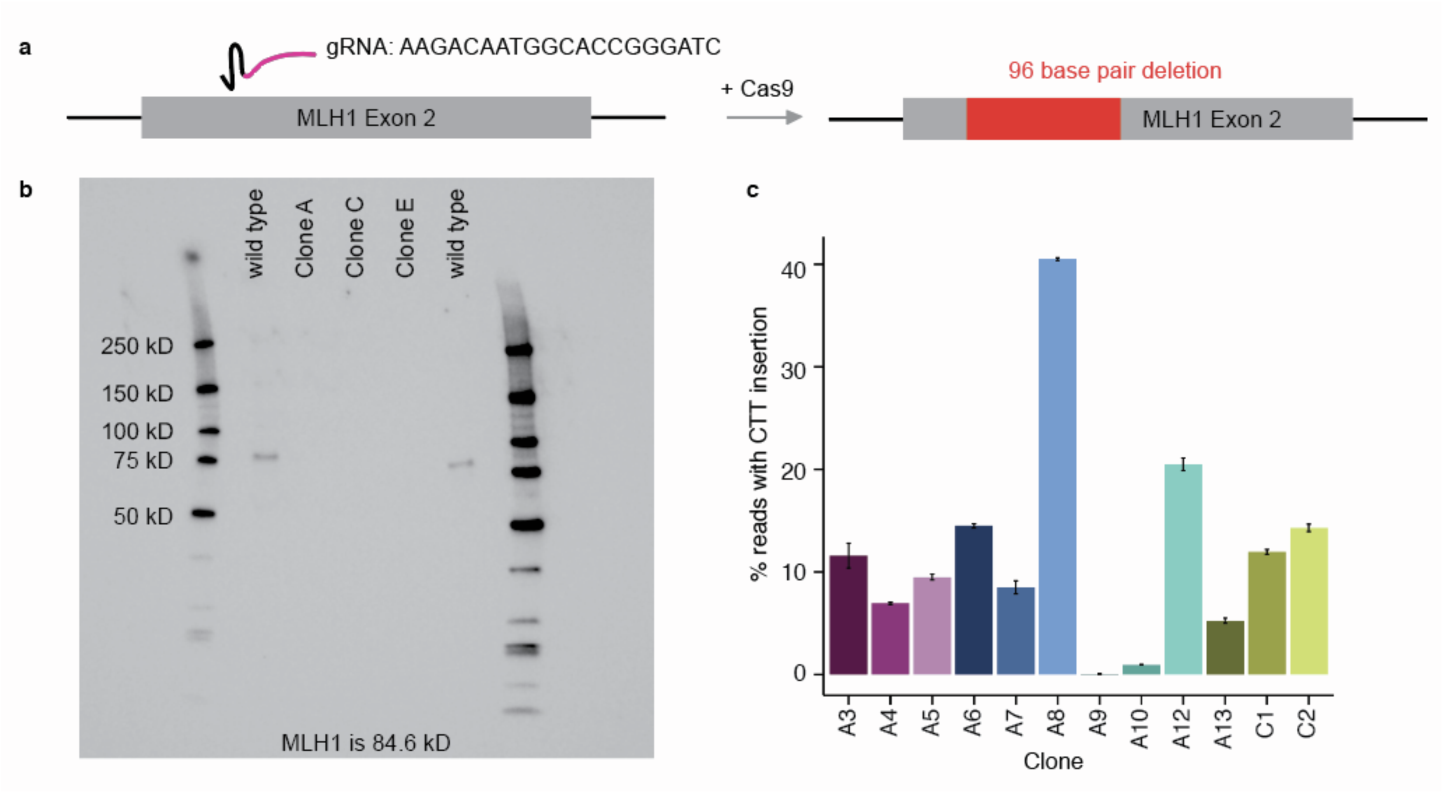

Figure S2 | Improvements to prime editing efficiency via an *MLH1* knockout. Related to STAR methods.

a) A single gRNA (in the pSpCas9(BB)-2A-Puro vector) targeting exon 2 in *MLH1* was transfected into PC-9. This knockout led to a 96 base pair deletion in both copies of *MLH1* in PC-9 cells. **b)** Western blot analysis of three PC-9 knockout clones. All three clones (clones A, C, and E) show complete loss of the MLH1 protein. Wild type cells were run in parallel (lanes 1 and 5) and show presence of the MLH1 protein. **c)** 12 monoclonal *MLH1*ko-PEmax-PC-9 cell lines were tested for insertion efficiency of a trinucleotide CTT insertion at the *HEK3* locus.

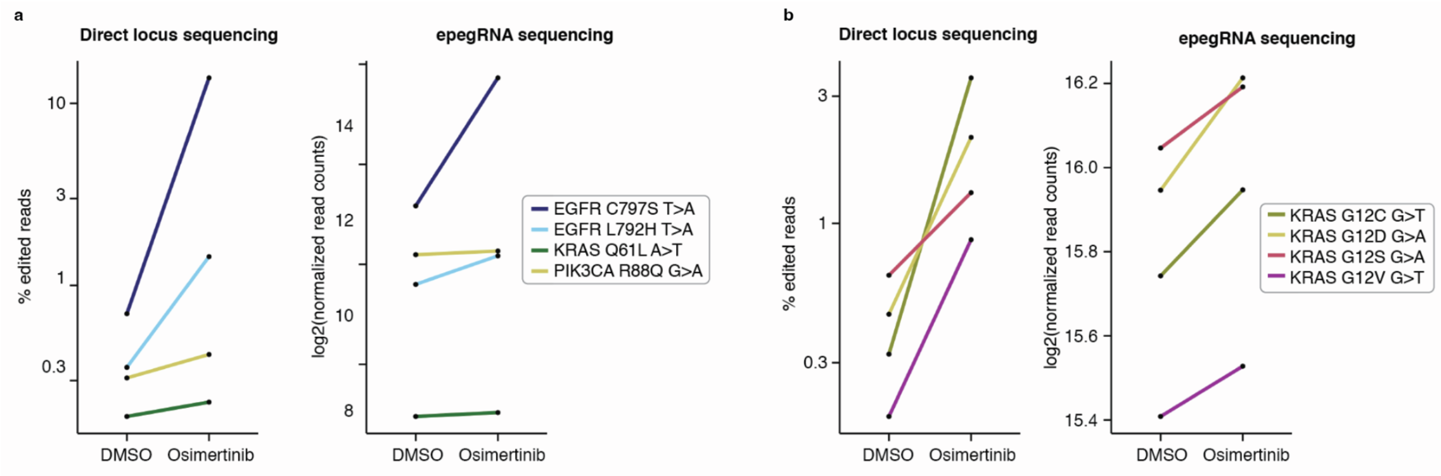

Figure S3 | Side-by-side sequencing of genomic loci and epegRNAs targeting those loci. Related to Figure 2.

a) Left: Percent edited reads by direct locus sequencing results in the no drug (DMSO) and osimertinib drug treatment arms. Right: log2 normalized read counts 339 of the epegRNA that programmed the specified edits. **b)** Same as a) but for a different set of targets.

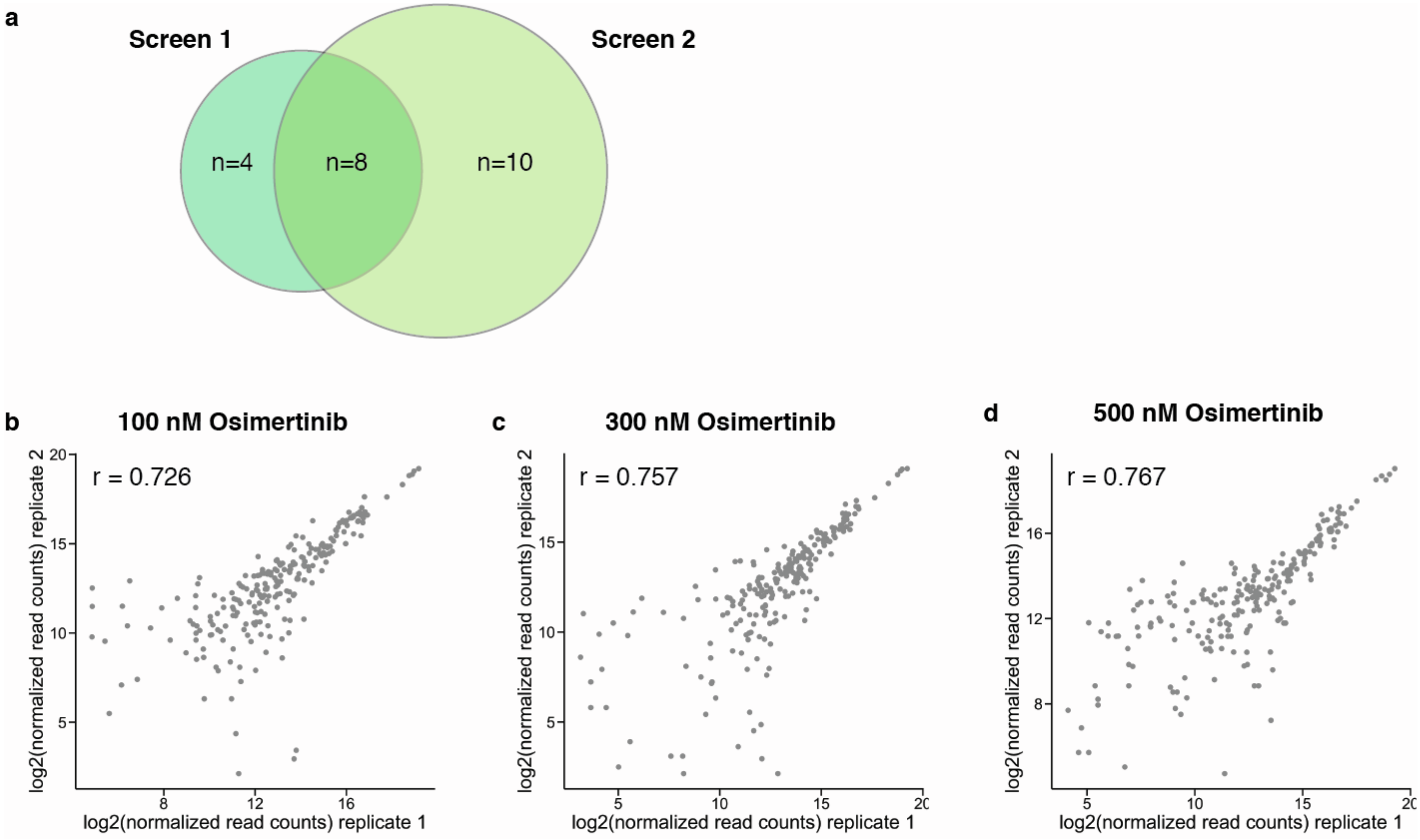

Figure S4 | Overlap of resistant variants between 121 epegRNA screens and replicate correlation of second 121 epegRNA screen. Related to Figure 2.

a) Overlap of hits between the first and second 121 epegRNA screens. **b)** Replicate correlation shown for 121 epegRNA screen at 100 nM osimertinib harvested at seven timepoints (days 3, 7, 10, 14, 17, 21, and 24). Each data point represents a single epegRNA at a single timepoint. **c)** Replicate correlation shown for 121 epegRNA screen at 300 nM osimertinib harvested at seven timepoints (days 3, 7, 10, 14, 17, 21, and 24). Each data point represents a single epegRNA at a single timepoint. **d)** Replicate correlation shown for 121 epegRNA screen at 500 nM osimertinib harvested at seven timepoints (days 3, 7, 10, 14, 17, 21, and 24). Each data point represents a single epegRNA at a single timepoint.

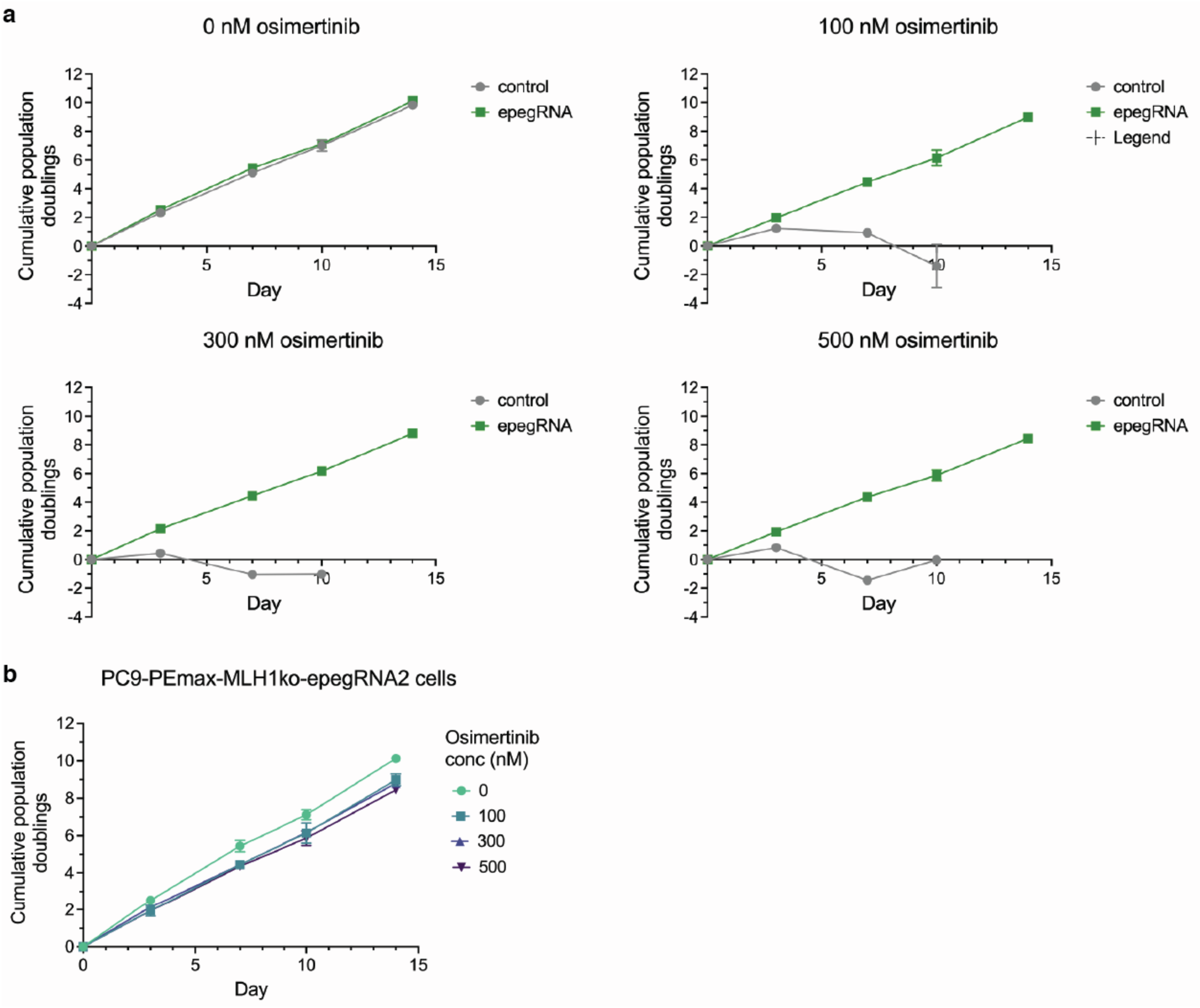

Figure S5 | PC-9 cell drug dosing experiments with varying concentrations of osimertinib. Related to STAR methods.

a) PC-9 cells with no integrated pegRNA ("control") or a pegRNA programming the EGFR C797S T>A osimertinib resistance mutation ("epegRNA"). Cells were treated with 0, 100, 300, and 500 nM osimertinib and cell population doublings were tracked over a period of 14 days. Data shown are the mean ± standard deviation of two biological replicates. **b)** Same data as in **a)**. Cell population doublings of osimertinib resistant, EGFR C797S T>A harboring cells in 0, 100, 300, and 500 nM osimertinib.

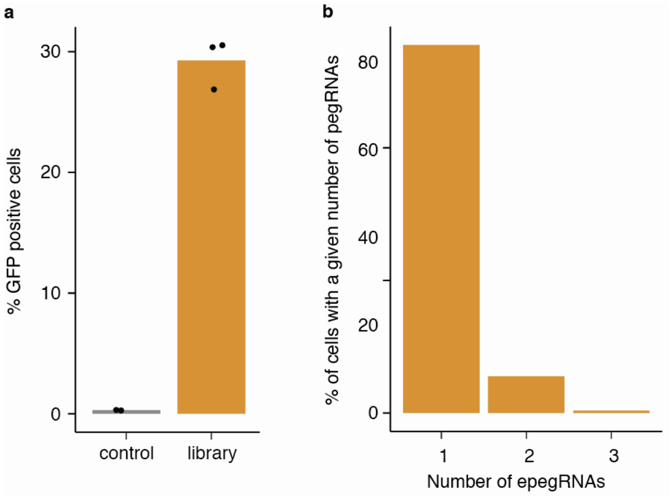

Figure S6 | Lentiviral transduction of 3,825 epegRNA library for scaled screen. Related to STAR methods.

a) *MLH1*ko-PEmax-PC-9 cells were analyzed by fluorescence activated cells sorting (FACS) to analyze the percentage of GFP+ cells following lentiviral transduction of the epegRNA library (GFP is expressed off the lentiviral epegRNA vector). Control cells were not transduced, and the library cells were transduced with the epegRNA library. The three data points represent three independent transduction replicates. **b)** Plot showing the percentage of cells harboring 1, 2, and 3 epegRNAs based off of the achieved MOI (~0.35) assuming a Poisson distribution of the number of integrations per cell.

a

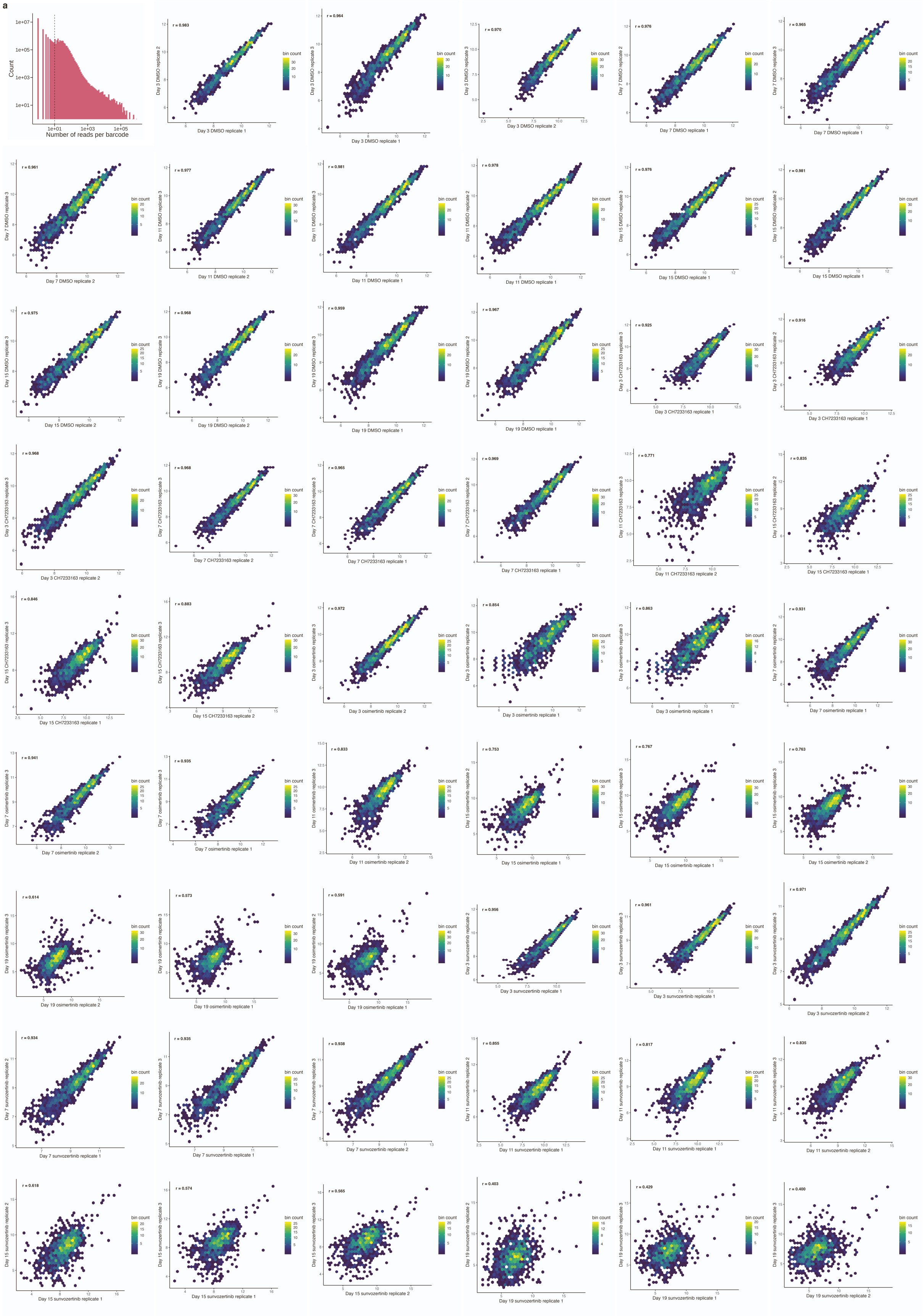
1e+07
1e+05
1e+03
1e+01
Count
1e+01
1e+03
1e+05
Number of reads per barcode
r = 0.983
Day 3 DMSO replicate 2
Day 3 DMSO replicate 1
r = 0.964
Day 3 DMSO replicate 3
Day 3 DMSO replicate 1
r = 0.970
Day 3 DMSO replicate 3
Day 3 DMSO replicate 2
r = 0.976
Day 7 DMSO replicate 2
Day 7 DMSO replicate 1
r = 0.965
Day 7 DMSO replicate 3
Day 7 DMSO replicate 1
r = 0.961
Day 7 DMSO replicate 3
Day 7 DMSO replicate 2
r = 0.977
Day 11 DMSO replicate 3
Day 11 DMSO replicate 2
r = 0.981
Day 11 DMSO replicate 3
Day 11 DMSO replicate 1
r = 0.978
Day 11 DMSO replicate 2
Day 11 DMSO replicate 1
r = 0.976
Day 15 DMSO replicate 3
Day 15 DMSO replicate 1
r = 0.981
Day 15 DMSO replicate 2
Day 15 DMSO replicate 1
r = 0.975
Day 15 DMSO replicate 3
Day 15 DMSO replicate 2
r = 0.968
Day 19 DMSO replicate 3
Day 19 DMSO replicate 2
r = 0.959
Day 19 DMSO replicate 3
Day 19 DMSO replicate 1
r = 0.967
Day 19 DMSO replicate 2
Day 19 DMSO replicate 1
r = 0.925
Day 3 CH7233163 replicate 3
Day 3 CH7233163 replicate 1
r = 0.916
Day 3 CH7233163 replicate 2
Day 3 CH7233163 replicate 1
r = 0.968
Day 3 CH7233163 replicate 3
Day 3 CH7233163 replicate 2
r = 0.968
Day 7 CH7233163 replicate 3
Day 7 CH7233163 replicate 2
r = 0.965
Day 7 CH7233163 replicate 3
Day 7 CH7233163 replicate 1
r = 0.969
Day 7 CH7233163 replicate 2
Day 7 CH7233163 replicate 1
r = 0.771
Day 11 CH7233163 replicate 3
Day 11 CH7233163 replicate 2
r = 0.835
Day 15 CH7233163 replicate 2
Day 15 CH7233163 replicate 1
r = 0.846
Day 15 CH7233163 replicate 3
Day 15 CH7233163 replicate 1
r = 0.883
Day 15 CH7233163 replicate 3
Day 15 CH7233163 replicate 2
r = 0.972
Day 3 osimertinib replicate 3
Day 3 osimertinib replicate 2
r = 0.854
Day 3 osimertinib replicate 2
Day 3 osimertinib replicate 1
r = 0.863
Day 3 osimertinib replicate 3
Day 3 osimertinib replicate 1
r = 0.931
Day 7 osimertinib replicate 2
Day 7 osimertinib replicate 1
r = 0.941
Day 7 osimertinib replicate 3
Day 7 osimertinib replicate 2
r = 0.935
Day 7 osimertinib replicate 3
Day 7 osimertinib replicate 1
r = 0.833
Day 11 osimertinib replicate 3
Day 11 osimertinib replicate 2
r = 0.753
Day 15 osimertinib replicate 2
Day 15 osimertinib replicate 1
r = 0.767
Day 15 osimertinib replicate 3
Day 15 osimertinib replicate 1
r = 0.763
Day 15 osimertinib replicate 3
Day 15 osimertinib replicate 2
r = 0.614
Day 19 osimertinib replicate 3
Day 19 osimertinib replicate 2
r = 0.573
Day 19 osimertinib replicate 3
Day 19 osimertinib replicate 1
r = 0.591
Day 19 osimertinib replicate 2
Day 19 osimertinib replicate 1
r = 0.956
Day 3 sunvozertinib replicate 2
Day 3 sunvozertinib replicate 1
r = 0.961
Day 3 sunvozertinib replicate 3
Day 3 sunvozertinib replicate 1
r = 0.971
Day 3 sunvozertinib replicate 3
Day 3 sunvozertinib replicate 2
r = 0.934
Day 7 sunvozertinib replicate 2
Day 7 sunvozertinib replicate 1
r = 0.935
Day 7 sunvozertinib replicate 3
Day 7 sunvozertinib replicate 1
r = 0.938
Day 7 sunvozertinib replicate 3
Day 7 sunvozertinib replicate 2
r = 0.855
Day 11 sunvozertinib replicate 2
Day 11 sunvozertinib replicate 1
r = 0.817
Day 11 sunvozertinib replicate 3
Day 11 sunvozertinib replicate 1
r = 0.835
Day 11 sunvozertinib replicate 3
Day 11 sunvozertinib replicate 2
r = 0.618
Day 15 sunvozertinib replicate 2
Day 15 sunvozertinib replicate 1
r = 0.574
Day 15 sunvozertinib replicate 3
Day 15 sunvozertinib replicate 1
r = 0.565
Day 15 sunvozertinib replicate 3
Day 15 sunvozertinib replicate 2
r = 0.403
Day 19 sunvozertinib replicate 2
Day 19 sunvozertinib replicate 1
r = 0.429
Day 19 sunvozertinib replicate 3
Day 19 sunvozertinib replicate 1
r = 0.400
Day 19 sunvozertinib replicate 3
Day 19 sunvozertinib replicate 2
bin count

Figure S7 | Barcode read count cutoff and replicate correlations in scaled screen. Related to STAR methods.

a) Histogram showing the number of sequencing reads per barcode. A read cutoff of 10 reads per barcode was used for all analyses. **b)** Replicate correlation plots of log2(normalized read counts per edit ID) of three independent transduction replicates in the three drug screens.

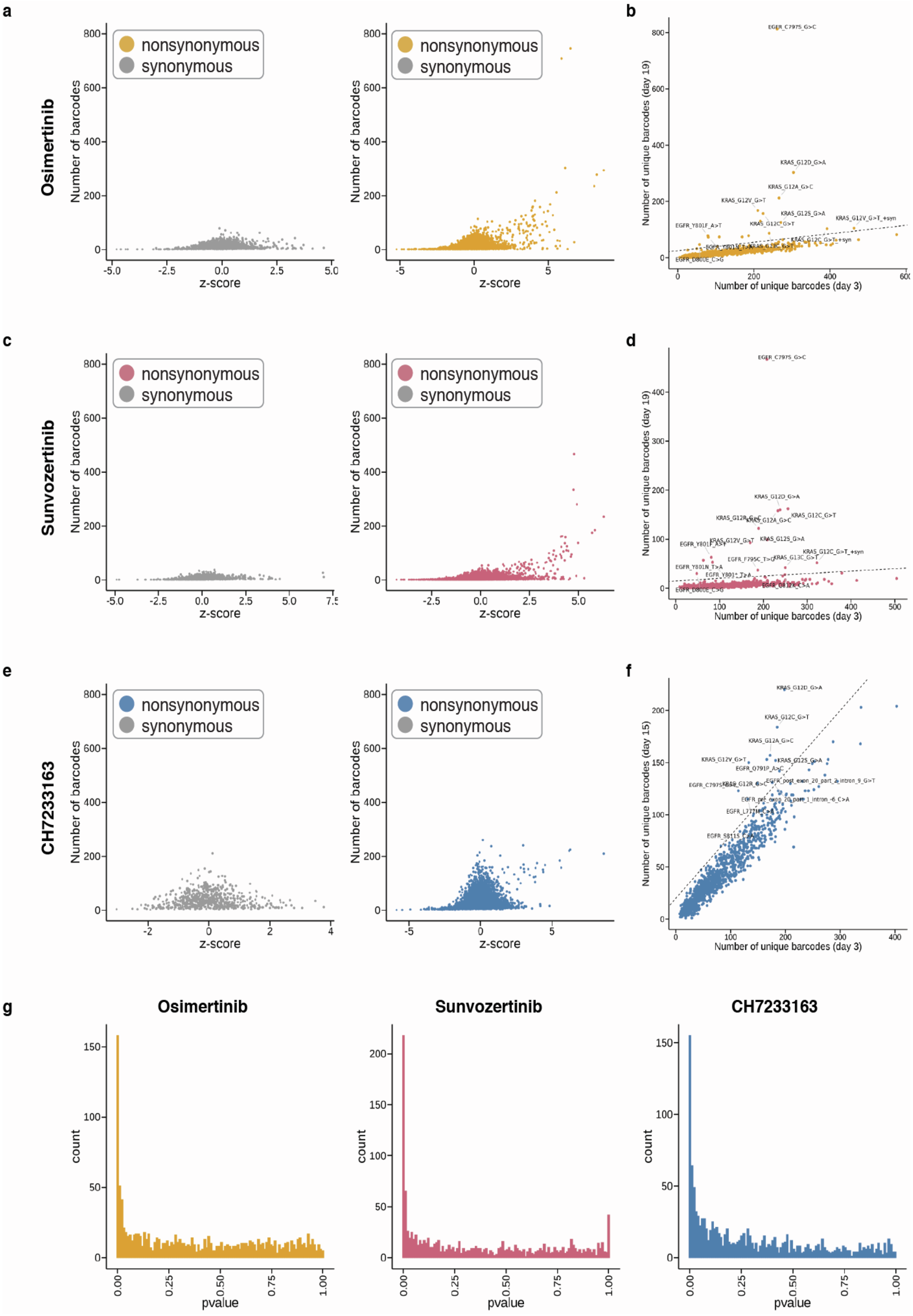
a
b
c
d
e
f
g
Osimertinib
Sunvozertinib
CH7233163
nonsynonymous
synonymous
Number of barcodes
z-score
Number of unique barcodes (day 19)
Number of unique barcodes (day 3)
Number of unique barcodes (day 15)
count
pvalue

Figure S8 | Z-score, barcode count, and p-value statistics from 3,825 epegRNA drug screens. Related to Figure 3.

a) Z-score and barcode counts plotted for day 15 and day 19 data (combined) for all three replicates for the osimertinib screen. Left: synonymous variants, right: nonsynonymous variants. **b)** Unique barcode count correlation plot between day 3 and day 19 of the osimertinib screen. Variants that fall above the diagonal (y = 0.15x + 25) are labeled. **c)** Z-score and barcode counts plotted for day 15 and day 19 data (combined) for all three replicates for the sunvozertinib screen. Left: synonymous variants, right: nonsynonymous variants. **d)** Unique barcode count correlation plot between day 3 and day 19 of the sunvozertinib screen. Variants that fall above the diagonal (y = 0.05x + 15) are labeled. **e)** Z-score and barcode counts plotted for day 15 and day 19 data (combined) for all three replicates for the CH7233163 screen. Left: synonymous variants, right: nonsynonymous variants. **f)** Unique barcode count correlation plot between day 3 and day 15 of the CH7233163 screen. Variants that fall above the diagonal (y = 0.6x + 20) are labeled. **g)** p-value distributions from DESeq2 differential pegRNA abundance testing for the three drug screens.

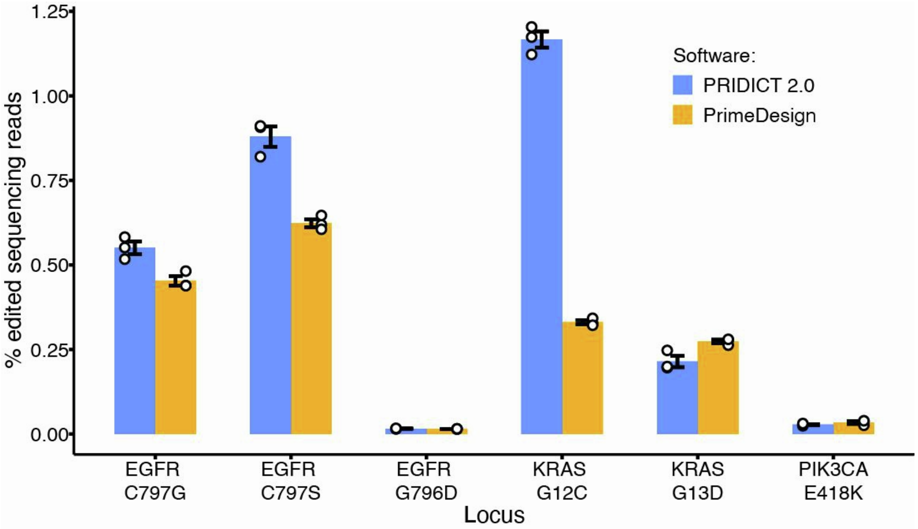

Figure S9 | Comparison of pegRNAs designed by PrimeDesign and PRIDICT 2.0. Related to STAR methods. Edit efficiencies of six different variants using pegRNAs designed with both PRIDICT 2.0 (blue) or PrimeDesign (yellow). Each bar represents the average of three replicates (denoted by points). Error bars represent ± one standard error of the mean.

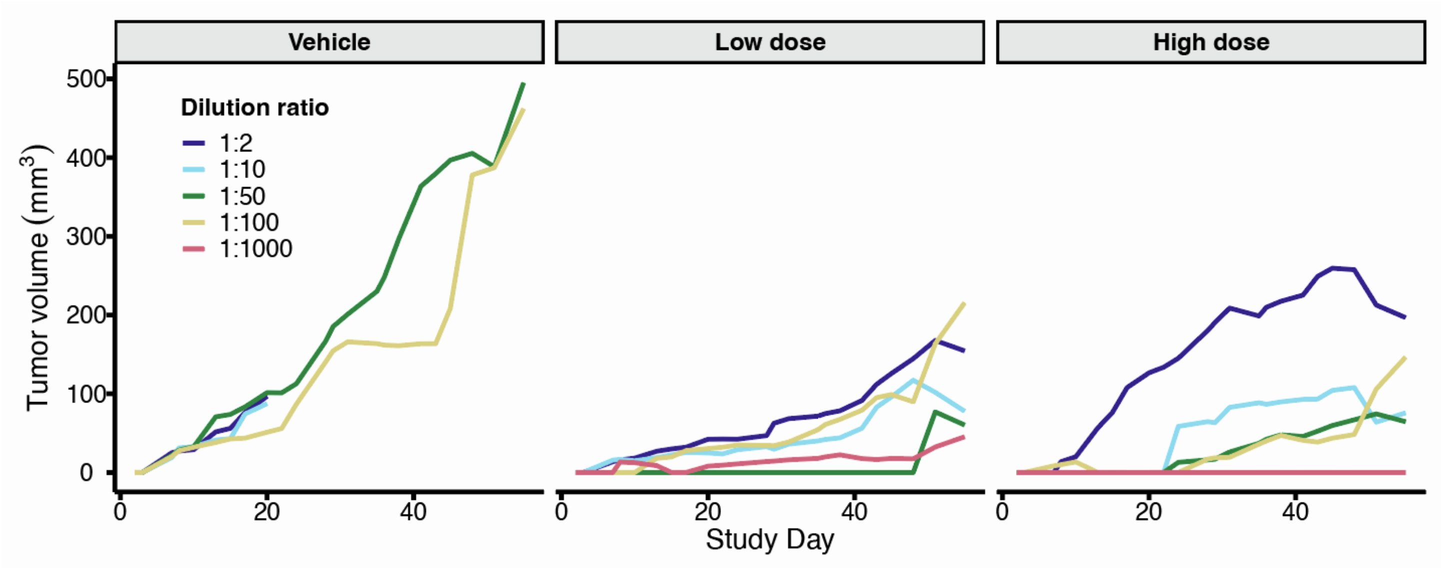

Figure S10 | Tumor volume over time in mice injected with mixtures of wild-type and EGFR C797S mutant PC-9 cells. Related to Figure 4.

Visualization of tumor volume over time in mice injected with mixtures of wild-type and EGFR C797S mutant PC-9 cells mixed at the indicated ratios. Plots are faceted by treatment group, including vehicle (DMSO control), low dose osimertinib (5 mg/kg) and high dose osimertinib (20 mg/kg).

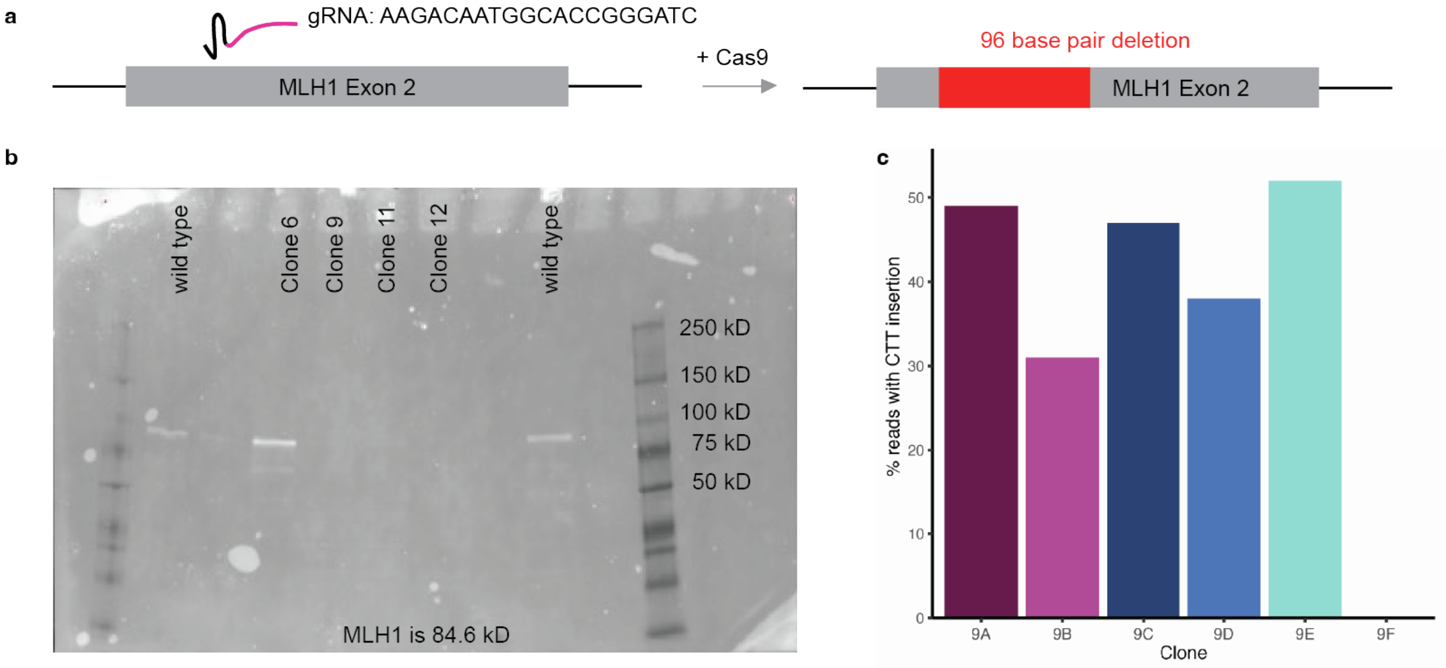

Figure S11 | Generation of an *MLH1* knockout A-375 cell line. Related to STAR methods.

a) A single gRNA (in the pSpCas9(BB)-2A-Puro vector) targeting exon 2 in *MLH1* was transfected into A-375 cells. This knockout leads to a 96 base pair deletion in both copies of *MLH1* in A-375 cells when successful. **b)** Western blot analysis of four A-375 knockout clones. Three of four clones (clones 9, 11, and 12) show complete loss of the MLH1 protein. Wild type cells were run in parallel (lanes 1 and 6) and show presence of the MLH1 protein. **c)** 6 monoclonal subclones of Clone 9 were tested for insertion efficiency of a trinucleotide CTT insertion at the *HEK3* locus. Clone 9E was used for all experiments.
